# Photic zone dynamics and environmental controls of pigment-derived phytoplankton size classes in the South-Eastern Black Sea

**DOI:** 10.1093/plankt/fbag063

**Published:** 2026-08-21

**Authors:** Ertuğrul Ağırbaş, Nazlı Genç

**Affiliations:** Faculty of Fisheries, Department of Marine Biology, Recep Tayyip Erdoğan University, 53100, Rize, Türkiye; Faculty of Fisheries, Department of Marine Biology, Recep Tayyip Erdoğan University, 53100, Rize, Türkiye

**Keywords:** phytoplankton size structure, HPLC pigment analysis, diagnostic pigments, subsurface chlorophyll maximum, coastal–offshore gradient, Black Sea

## Abstract

Understanding the environmental controls on phytoplankton is essential for predicting ecosystem responses to changing ocean conditions. This study investigated pigment-derived phytoplankton size classes (micro-, nano- and picophytoplankton) along a river–coastal–offshore gradient in the south-eastern Black Sea using high-performance liquid chromatography (HPLC) and Canonical Correspondence Analysis (CCA). Monthly sampling was conducted between May 2015 and April 2016 at three stations (0.5, 5 and 20 nautical miles offshore). Water samples collected throughout the photic zone were analyzed for chlorophyll-*a* and diagnostic pigments in relation to hydrography, nutrient availability and light conditions. Microphytoplankton contributed 8–91% of total phytoplankton biomass and dominated nutrient-rich, low-salinity river-influenced waters, whereas nanophytoplankton prevailed in coastal transitional zones and picophytoplankton reached up to 70% in oligotrophic offshore environments. CCA showed that phytoplankton size structure was primarily governed by cross-shelf gradients in nutrient availability, salinity and depth, with chlorophyll-*a* associated with microphytoplankton dominance and temperature and stratification favoring smaller size classes. These findings demonstrate strong bottom–up control of phytoplankton size structure and emphasize the importance of cross-shelf environmental gradients in regulating ecosystem organization. The offshore shift toward smaller phytoplankton fractions suggests reduced trophic transfer efficiency and increasing reliance on microbial pathways for carbon cycling under enhanced water-column stratification.

## INTRODUCTION

Phytoplankton constitute the primary producers of pelagic ecosystems and are responsible for a substantial proportion of global organic carbon production. They form the energetic foundation of marine food webs by mediating energy transfer from primary producers to higher trophic levels, including zooplankton, fish, seabirds and marine mammals (Tait and Dipper, 2001). Owing to their exceptionally high photosynthetic capacity, phytoplankton contribute approximately half of global primary production (Falkowski and Raven, 2007; Boyce *et al.,* 2010) and represent the dominant source of biological production in marine environments (Mackas, 2011). Beyond their fundamental trophic role, phytoplankton exert a critical influence on global biogeochemical cycles by regulating air–sea CO₂ exchange and carbon fixation in surface waters, thereby contributing to oceanic pH balance and the efficiency of the biological carbon pump. It has been estimated that the oceans absorb nearly one-third of anthropogenic CO₂ emissions, significantly mitigating the greenhouse effect (Takahashi *et al.,* 2002; Sabine and Feely, 2007). Consequently, any shift in phytoplankton community structure can directly influence pelagic ecosystem productivity and biogeochemical functioning (Nagata *et al.,* 1996).

Traditionally, phytoplankton studies have relied on microscopic techniques involving taxonomic identification based on morphological characteristics, enumeration of cell abundance and biomass estimation (Utermöhl, 1958; Booth, 1993; Eker-Develi *et al.,* 2008). However, this approach is time-consuming, strongly dependent on taxonomic expertise and often constrained by sample processing limitations, which restrict the number of samples that can be analysed. Furthermore, very small phytoplankton groups such as picoplankton (≤2 μm), which lack distinctive morphological features, are difficult to identify using conventional microscopy (Mackey *et al.,* 1996). These limitations reduce the accuracy of size-fraction quantification, particularly in oligotrophic and stratified systems where small phytoplankton dominate.

Chlorophyll-*a* has long been used as a proxy for phytoplankton biomass and distribution, as well as for assessing physiological status in marine ecosystems. As the primary photosynthetic pigment, chlorophyll-*a* captures light energy, while accessory pigments enable phytoplankton to utilize different wavelengths and maintain photosynthetic activity across depth gradients (Yücel, 2017). Although spectrophotometric and fluorometric techniques have been widely applied for pigment measurements, these methods are subject to spectral interference and degradation-related biases, which can compromise accuracy (Gibb *et al.,* 2001). In contrast, high-performance liquid chromatography (HPLC) provides a more precise and reliable approach for identifying chlorophyll-*a* and diagnostic pigments associated with specific phytoplankton groups (Mantoura and Llewellyn, 1983; Jeffrey and Vesk, 1997). Importantly, HPLC-derived pigment signatures enable the quantitative reconstruction of phytoplankton functional groups and size classes, offering a robust alternative to morphology-based methods. This approach is particularly advantageous in oligotrophic environments, where low biomass and dominance of small cells require high-resolution analytical techniques (Li *et al.,* 1993; Partensky *et al.,* 1993; Bell and Kalff, 2001).

As in other marine ecosystems, the structure and functioning of phytoplankton communities in the Black Sea are closely linked to environmental variability, making them effective indicators of ecosystem status and change (Yunev *et al.,* 2002). Despite the high productivity of the Black Sea, most previous studies have focused on microscopic cell counts and taxonomic composition, predominantly conducted by Russian and Romanian research groups. Along the Anatolian coast, studies have generally been limited to seasonal or regional monitoring programs aimed at identifying phytoplankton species across broad spatial scales (Agirbas, 2010; Ağırbaş, 2016). However, investigations specifically addressing pigment-based phytoplankton size-class distributions remain scarce, particularly in the south-eastern Black Sea, where hydrographic variability and coastal inputs strongly influence ecosystem dynamics (Ağırbaş, 2016).

Pigment-based approaches have been widely applied in marine systems worldwide (Wright and Jeffrey, 1987; Gieskes, 1991; Millie *et al.,* 1993; Jeffrey and Vesk, 1997; Gibb *et al.,* 2000, 2001; Barlow *et al.,* 2002, 2004; Aiken *et al.,* 2009; Agirbas *et al*., 2015). In the Black Sea, however, most studies have focused primarily on chlorophyll-*a* measurements (Yılmaz *et al.,* 1998; Kopelevich *et al.,* 2002; Yunev *et al.,* 2002; Eker-Develi and Kideys, 2003; Yılmaz *et al.,* 2006,), and comprehensive analyses integrating diagnostic pigments with size-class partitioning remain limited. Although the application of HPLC-based pigment analysis has increased in recent years (Ediger *et al.,* 2006; Agirbas, 2010; Eker-Develi *et al.,* 2012; Koca, 2014; Türkmen, 2016; Agirbas *et al*., 2017), a clear understanding of size-structured phytoplankton dynamics across environmental gradients is still lacking.

Over the past three decades, numerous studies have reported a shift toward smaller phytoplankton size classes in the Black Sea. The euphotic zone provides an ideal setting for pigment-based investigations; however, studies focusing specifically on size–class dynamics along the Anatolian coast remain limited. This knowledge gap restricts our ability to resolve how environmental gradients regulate phytoplankton size structure and to predict ecosystem-level consequences for carbon cycling and fisheries’ productivity. Therefore, this study aims to investigate the temporal and spatial variability of pigment-based phytoplankton size structure within the photic zone of the south-eastern Black Sea using HPLC analysis, with a particular focus on quantifying the relative contributions of micro-, nano- and picophytoplankton and their responses to seasonal and cross-shelf environmental gradients. In this context, it is hypothesized that small-sized phytoplankton (nano- and picophytoplankton) dominate under stratified and nutrient-depleted conditions, whereas microphytoplankton prevail during nutrient-rich periods; that phytoplankton size structure exhibits pronounced seasonal variability driven by changes in stratification and nutrient availability; that spatial gradients between coastal and offshore waters significantly regulate size–class composition through differences in nutrient supply; and that pigment-based HPLC analysis reveals a higher relative contribution of small phytoplankton fractions compared to traditional microscopy-based approaches, reflecting an ongoing shift toward smaller size classes associated with environmental change in the south-eastern Black Sea.

## MATERIALS AND METHODS

### Study area and samplings

The Black Sea is a semi-enclosed basin characterized by strong vertical stratification and complex hydrographic dynamics. The south-eastern sector, influenced by riverine inputs, coastal circulation and seasonal stratification, exhibits pronounced variability in nutrient availability and primary productivity, which, in turn, regulates phytoplankton distribution and composition. The study area is characterized by a relatively narrow continental shelf and dynamic circulation patterns driven by the cyclonic Rim Current system. Seasonal stratification and light attenuation generate distinct vertical gradients within the photic zone, creating suitable conditions for investigating size-structured phytoplankton dynamics.

**Fig. 1:**
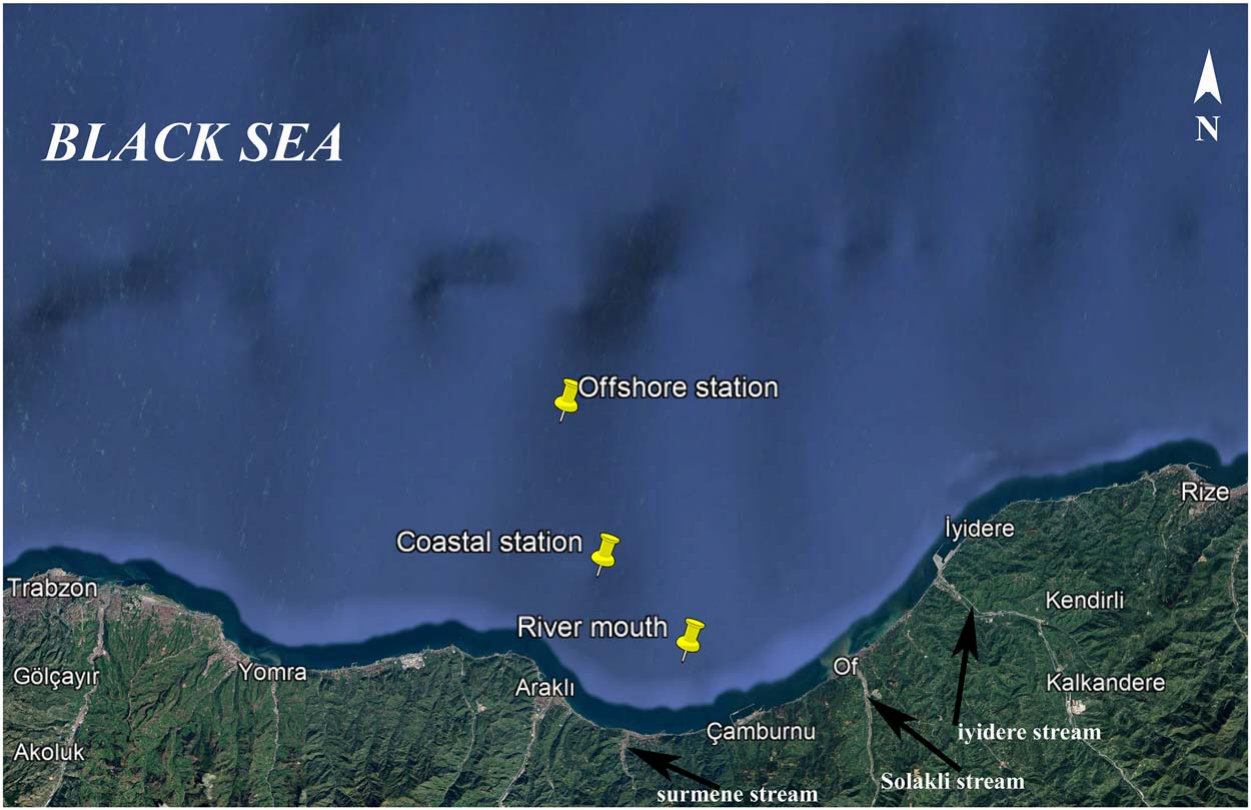
Map of the south-eastern Black Sea showing the sampling stations.

Sampling was conducted at three stations representing distinct hydrographic regimes along a coastal–offshore transect in the south-eastern Black Sea. Stations were located at 0.5 (river mouth), 5 (coastal) and 20 (offshore) nautical miles from the coastline and were sampled monthly between May 2015 and April 2016 (Fig. 1), allowing the assessment of both seasonal and spatial variability. Seawater samples were collected from multiple depths (surface, 10, 25 and 50 m) within the photic zone using Niskin bottles mounted on a conductivity–temperature–depth (CTD) rosette system. Sampling depths were selected to capture vertical variability within the photic layer and to resolve depth-dependent changes in phytoplankton structure. Sampling at fixed depths across all stations also ensured consistency among seasonal comparisons and multivariate analyses.

Hydrographic parameters, including temperature, salinity and depth, were recorded using a SeaBird SBE-19 Plus CTD profiler. *In situ* chlorophyll fluorescence was measured using a WETLAB fluorometer integrated into the CTD system. Photosynthetically active radiation (PAR) measurements were conducted using a Li-Cor underwater PAR sensor system (Li-193 SA spherical quantum sensor, Li-1 400 data logger and Li-190SAT surface sensor). The euphotic zone depth was defined as the depth receiving 1% of surface PAR. Although the euphotic depth occasionally occurred shallower than 50 m, water samples collected at 50 m were retained for pigment analyses, phytoplankton size–class calculations and Canonical Correspondence Analysis (CCA). These samples were included to characterize the lower photic–sub-euphotic transition and to provide a more comprehensive assessment of the vertical distribution of phytoplankton pigments and associated environmental gradients.

### HPLC and pigment analyses

Pigment analyses were performed using HPLC following Mantoura and Llewellyn (1983). Seawater samples (1 L) were filtered under low vacuum pressure (<0.5 atm) through 47 mm GF/F filters and immediately stored in liquid nitrogen (−196°C) to prevent pigment degradation. Prior to analysis, filters were extracted in 5 mL of 90% HPLC-grade acetone using sonication (60 Hz, 1 min), followed by overnight extraction at 4°C in darkness. After extraction, samples were centrifuged at 3500 rpm for 10 min to remove particulate material. All procedures were conducted under low-light conditions to minimize photo-oxidation.

Pigment separation was performed using a SHIMADZU HPLC system equipped with a C8 column and diode-array detector (DAD). Extracts were filtered (0.2 μm), mixed with ammonium acetate buffer, and 100 μL was injected into a Thermo Hypersil MOS-2 C8 column (150 × 4.6 mm, 3 μm). Pigments were separated using a linear gradient with a dual mobile-phase system consisting of methanol–ammonium acetate (phase A) and 100% methanol (phase B) at a flow rate of 1 mL min^−1^. Chromatographic integration was performed using LC Solution software. Calibration was conducted using certified pigment standards, including chlorophylls and diagnostic pigments (Sigma; VKI, Denmark). Detection limits ranged between 0.005 and 0.007 μg L^−1^, and analytical reliability was ensured through external calibration using certified standards, calibration curves with coefficients of determination (*R*^2^ > 0.99) and routine verification of instrument performance throughout the analytical period.

### Determination of pigment-based phytoplankton size classes

The relative contribution of phytoplankton size classes was estimated following Vidussi *et al.* (2001) using diagnostic pigment analysis. The weighted sum of diagnostic pigments (ΣDPw) was calculated as:

*ΣDP_w_ = 1.41[Fuco] + 1.41[Perid] + 1.27[Hex-fuco] + 0.35[But-fuco] + 0.60[Allo] + 1.01[TChl b] + 0.86[Zea].*

The fractional contributions of phytoplankton size classes were calculated as:

*f_micro_ = (1.41[Fuco] + 1.41[Perid])/ΣDP_w_.*

*f_nano_ = (1.27[Hex-fuco] + 0.35[But-fuco] + 0.60[Allo])/ΣDP_w_.*

*f_pico_ = (1.01[TChl b] + 0.86[Zea])/ΣDP_w_.*

where diagnostic pigments represent taxon-specific markers used to partition phytoplankton biomass into functional size classes. These indices provide a quantitative framework for assessing size-structured phytoplankton dynamics and enable robust ecological interpretation of community composition.

### Nutrient analyses

Seawater samples were collected in acid-cleaned (HCl-rinsed) 100 mL bottles and filtered through 0.45 μm filters. Silicate samples were stored at 4°C, whereas nitrate, nitrite and phosphate samples were frozen until analysis. Nutrient concentrations (NO₃^−^, NO₂^−^, PO₄^3−^ and SiO₂) were determined using a SEAL AutoAnalyzer following standard colorimetric methods (Grasshoff *et al.,* 1999). Analytical accuracy was ensured using certified reference materials and internal standards.

### Statistical analyses

Prior to analysis, data were log-transformed where necessary to meet normality assumptions. Normality and homogeneity of variance were assessed using Shapiro–Wilk and Levene’s tests, respectively and appropriate parametric or non-parametric tests were applied accordingly. Differences among stations were evaluated using one-way analysis of variance (ANOVA), followed by Tukey’s HSD *post hoc* test when significant differences were detected. Detailed pairwise comparisons among stations are presented in Supplementary Table S6. Relationships between phytoplankton size classes and environmental variables were assessed using Pearson correlation analysis. In addition, Canonical Correspondence Analysis (CCA) was applied to identify the key environmental drivers structuring phytoplankton size composition (CANOCO 5.0). All statistical analyses were conducted using SigmaPlot 11.0, with significance set at *P* < 0.05.

## RESULTS

### Hydrographic characteristics of the water column

**Fig. 2:**
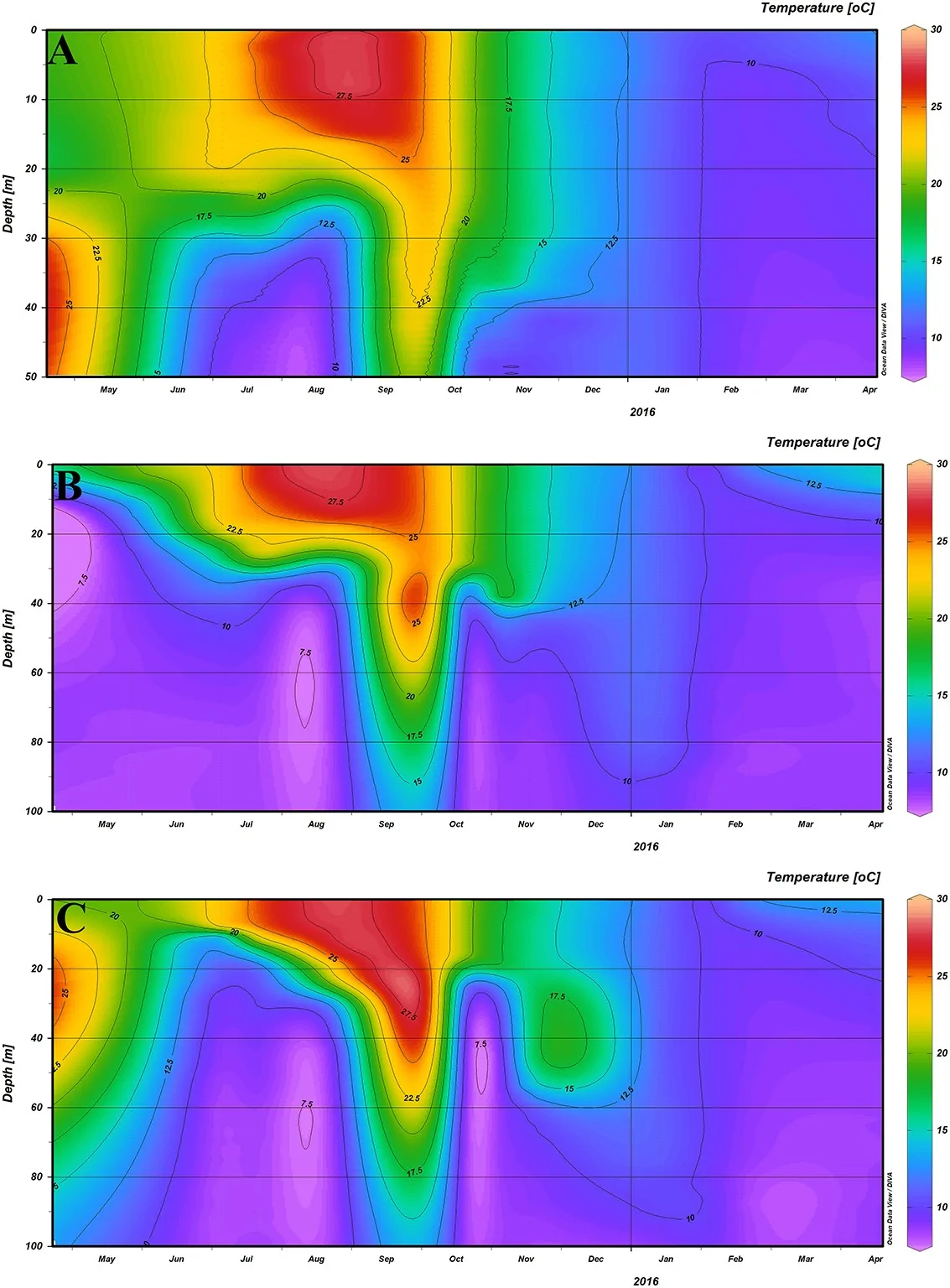
Spatio-temporal profiles of temperature along the study area: (a) river mouth station, (b) coastal station, (c) offshore station.

The hydrographic structure of the water column in the south-eastern Black Sea exhibited pronounced vertical stratification within the photic zone throughout the sampling period. Temperature profiles revealed a distinct thermocline separating warmer surface waters from cooler subsurface layers. Surface temperatures were consistently highest in the upper photic zone due to strong solar heating and limited vertical mixing, while temperature decreased progressively with depth, indicating a stable stratified water column (Fig. 2). The lowest sea surface temperature was recorded in February (9.7°C), whereas the highest temperature occurred in August (28.2°C). A seasonal thermocline began to develop in May and became more pronounced during July and August. With the onset of vertical mixing in December, the thermocline gradually weakened, and the water column exhibited a relatively homogeneous structure during the winter months (January–March). This seasonal pattern delineates two distinct hydrographic regimes: (i) a strongly stratified summer period with limited vertical mixing and (ii) a well-mixed winter period characterized by enhanced vertical exchange.

**Fig. 3:**
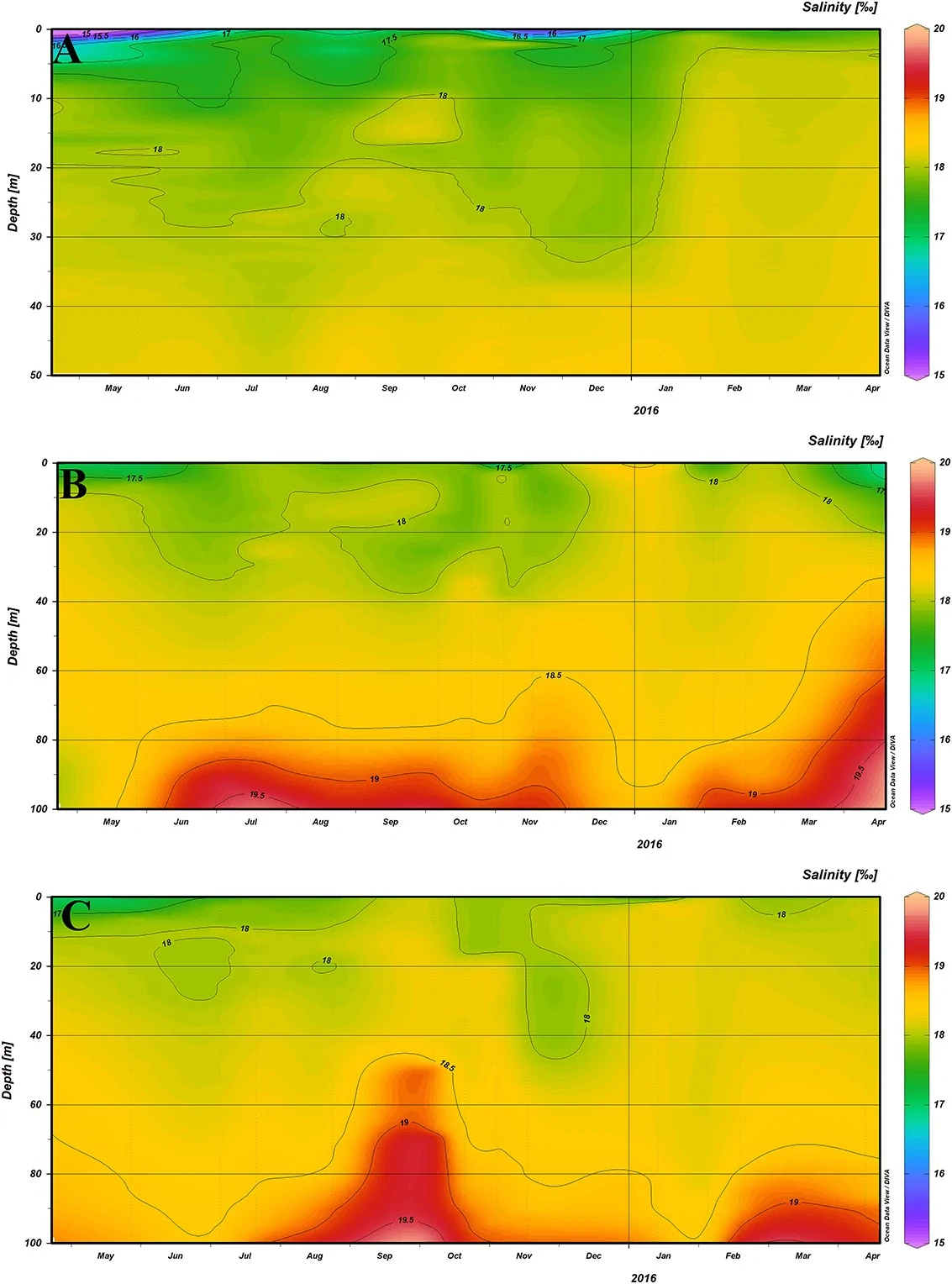
Spatio-temporal profiles of salinity along the study area: (a) river mouth station, (b) coastal station, (c) offshore station.

Salinity exhibited comparatively weaker vertical variability than temperature but showed consistent gradients associated with freshwater inputs and regional circulation. Lower salinity values were generally observed in surface layers, particularly at river mouth and coastal stations, reflecting the influence of river discharge and precipitation. In contrast, salinity increased with depth and stabilized in deeper photic layers, indicating the presence of more homogeneous subsurface water masses. Surface salinity values ranged between 16‰ and 18‰, increasing to 19–20‰ at depth (Fig. 3). Although moderate, this vertical salinity gradient indicates persistent freshwater influence in surface waters, contributing to stratification of the upper water column.

The combined temperature and salinity structure confirms the presence of a stratified water column that varies seasonally and spatially across the study area. Stratification was most intense during summer, while winter conditions were characterized by vertical homogenization. In addition, the spatial variability in salinity between nearshore and offshore stations reflects a clear coastal–offshore gradient, with lower salinity at river-influenced stations and more stable conditions offshore. These hydrographic patterns provide the physical basis for understanding variability in phytoplankton size structure, particularly in relation to seasonal stratification and cross-shelf gradients in water column properties.

### *In situ* fluorescence (chlorophyll-*a*)

**Fig. 4:**
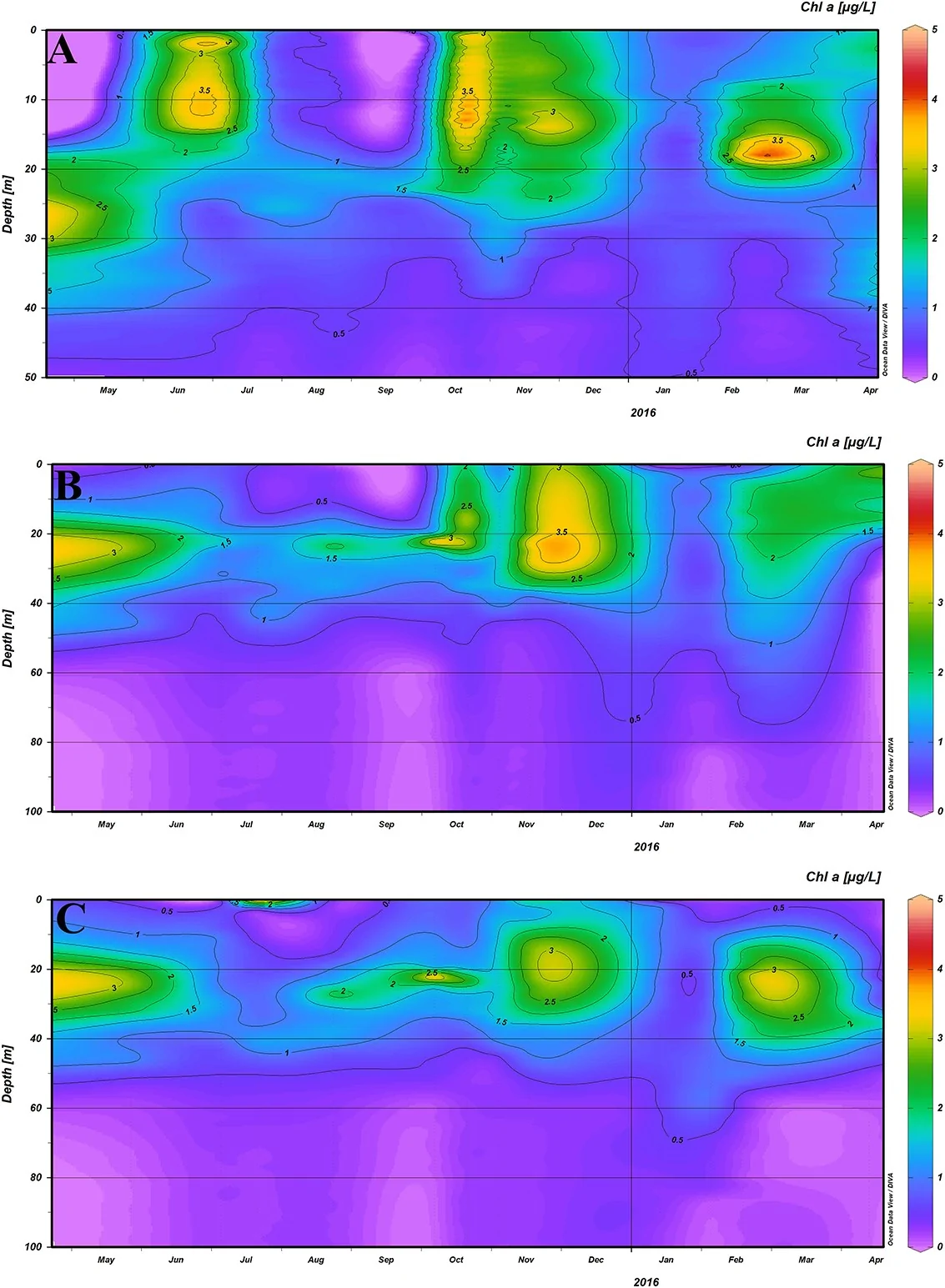
Spatio-temporal profiles of *in situ* Chl-*a* along the study area: (a) river mouth station, (b) coastal station, (c) offshore station.

Fluorescence-based chlorophyll-*a* concentrations showed pronounced vertical variability within the photic zone, indicating marked heterogeneity in phytoplankton biomass distribution. In most sampling stations, elevated chlorophyll-*a* concentrations were observed in the upper photic layer, reflecting enhanced phytoplankton biomass in surface waters where light availability is highest (Fig. 4). However, this vertical pattern exhibited clear seasonal variability and was not consistently confined to surface layers. In several profiles, a subsurface chlorophyll maximum (SCM) was detected within the section of the photic zone. This feature was particularly evident during stratified periods and varied in both intensity and depth across seasons. Below the SCM, chlorophyll-*a* concentrations generally decreased toward deeper photic layers, corresponding to reduced light availability. The presence of a distinct SCM and its seasonal variability indicate a vertically structured phytoplankton distribution within the water column.

Spatial differences in chlorophyll-*a* concentrations were also evident among stations. The river mouth station consistently exhibited higher chlorophyll-*a* values compared to coastal and offshore stations, while concentrations generally decreased along the coastal–offshore gradient. Within the photic zone, chlorophyll-*a* concentrations ranged between 0.19 μg L^−1^ (September) and 4.71 μg L^−1^ (October) at the river mouth station, 0.16 μg L^−1^ (April) and 3.74 μg L^−1^ (September) at the coastal station and 0.18 μg L^−1^ (June) and 3.70 μg L^−1^ (September) at the offshore station. Surface chlorophyll-*a* concentrations varied between 0.51–3.97 μg L^−1^ at the river mouth station, 0.16–2.47 μg L^−1^ at the coastal station and 0.18–3.04 μg L^−1^ at the offshore station. These results demonstrate a clear spatial gradient in chlorophyll-*a* distribution, with higher biomass in river-influenced waters and lower concentrations offshore. In addition, the occurrence and persistence of the SCM highlight the importance of vertical structure in shaping phytoplankton biomass distribution within the photic zone.

### Photosynthetically active radiation

**Fig. 5:**
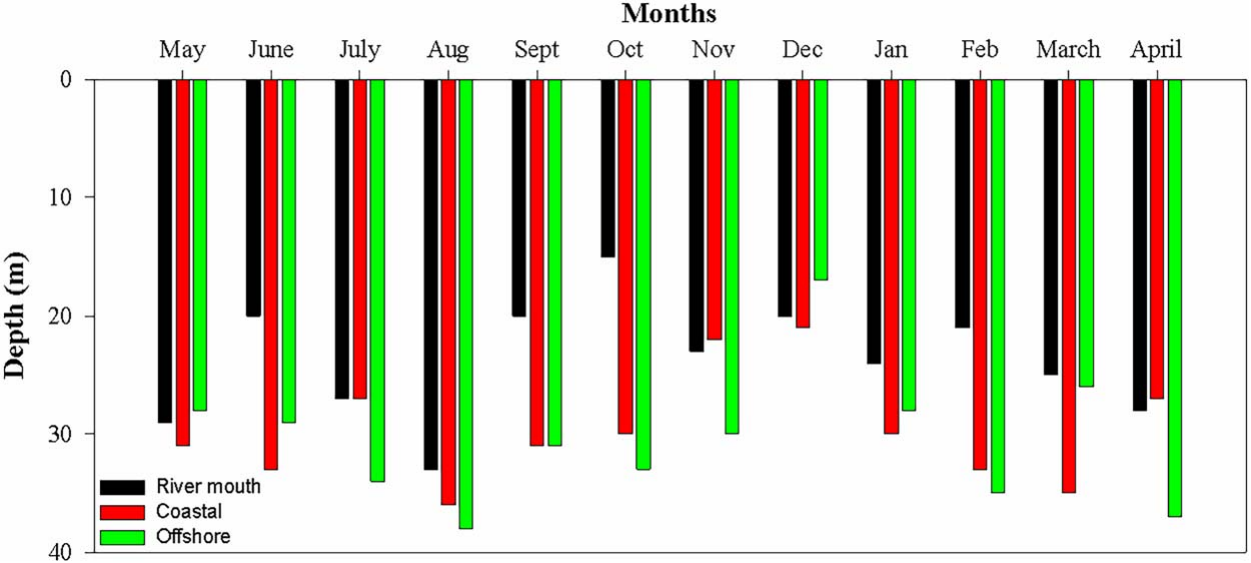
Photic zone depths determined from photosynthetically active radition (PAR) profiles in the three study stations through time.

PAR measurements conducted to determine the depth of the photic zone revealed clear spatial variability among sampling stations (Fig. 5). The photic zone depth ranged between 15–33 m at the river mouth station, 21–36 m at the coastal station and 17–38 m at the offshore station. This variability indicates differences in water transparency across the study area. The relatively shallower photic depth observed at the river mouth station reflects higher light attenuation, whereas deeper photic zones at the coastal and offshore stations indicate comparatively clearer water conditions.

Seasonally, photic depth exhibited systematic variability, with reduced light penetration during periods of elevated chlorophyll-*a* concentrations and increased surface productivity, and deeper light penetration during periods of lower biomass and enhanced water transparency. These seasonal changes in photic depth correspond to variations in light attenuation within the water column. The observed spatial and temporal patterns in PAR define the vertical extent of the photic zone and provide a physical framework for interpreting variations in phytoplankton distribution. In addition, the deeper photic zone observed at offshore stations is consistent with the occurrence of subsurface chlorophyll maxima described in the *In situ* fluorescence (Chlorophyll-*a*) section.

### Nutrient concentrations

**Fig. 6:**
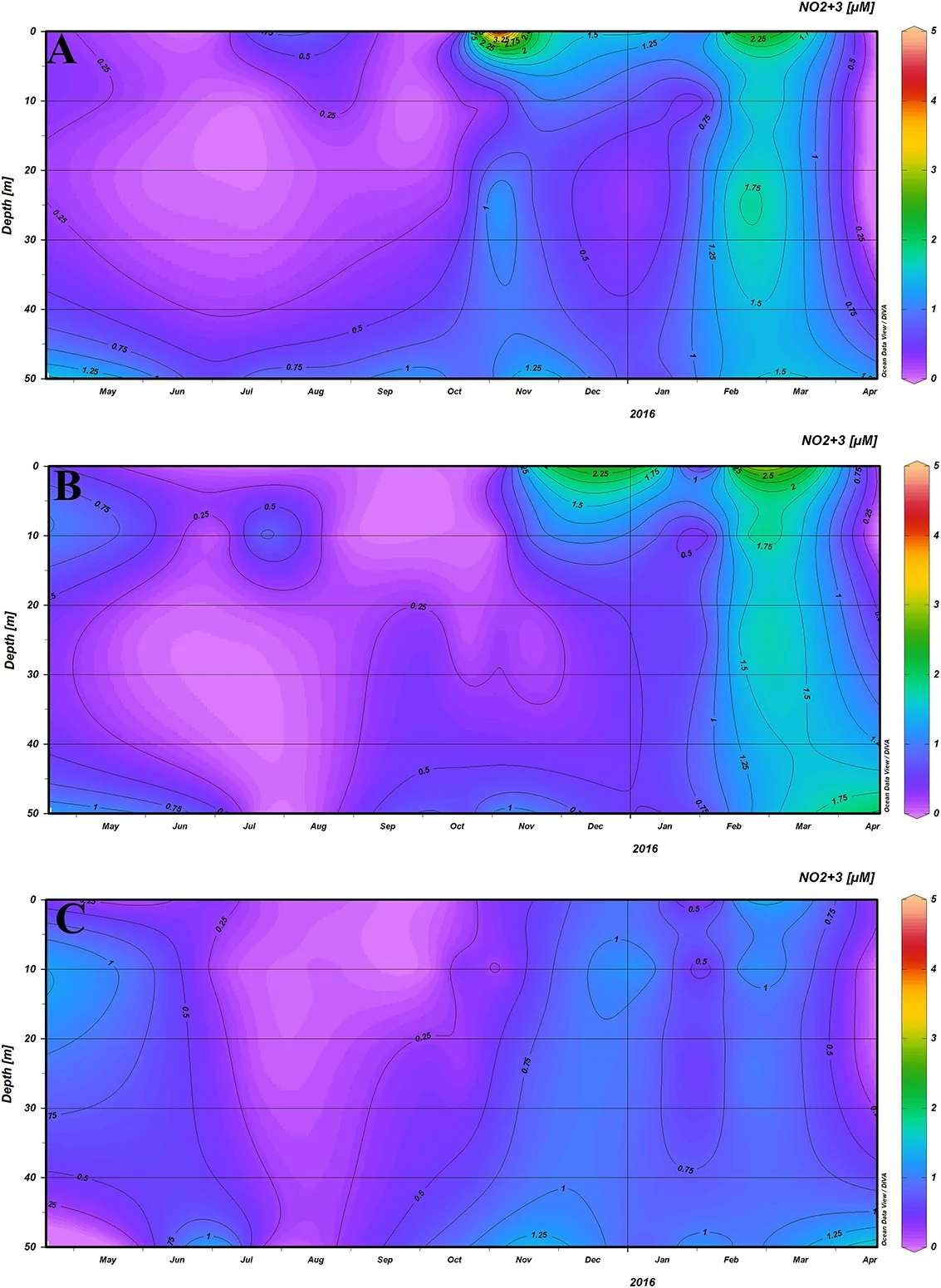
Spatio-temporal profiles of NO_2_ + NO_3_ concentrations along the study area: (a) river mouth station, (b) coastal station, (c) offshore station.

The concentrations of nitrite + nitrate (NO₂^−^ + NO₃^−^) measured within the photic zone showed significant spatial and temporal variability among sampling stations (ANOVA, *P* < 0.05). Throughout the study period, NO₂^−^ + NO₃^−^ concentrations remained generally below 5 μM. At the river mouth station, concentrations ranged from 0.01 μM (July) to 4.95 μM (November), consistently exceeding values recorded at coastal and offshore stations. At the coastal station, concentrations varied between 0.01 μM (July, 25 m) and 2.82 μM (March), whereas at the offshore station, values ranged between 0.01 μM (July) and 1.62 μM (April) (Fig. 6). These results indicate a clear decline in nitrogen concentrations along the coastal–offshore gradient. Seasonally, minimum concentrations were observed during summer months, while higher values occurred during autumn and winter.

**Fig. 7:**
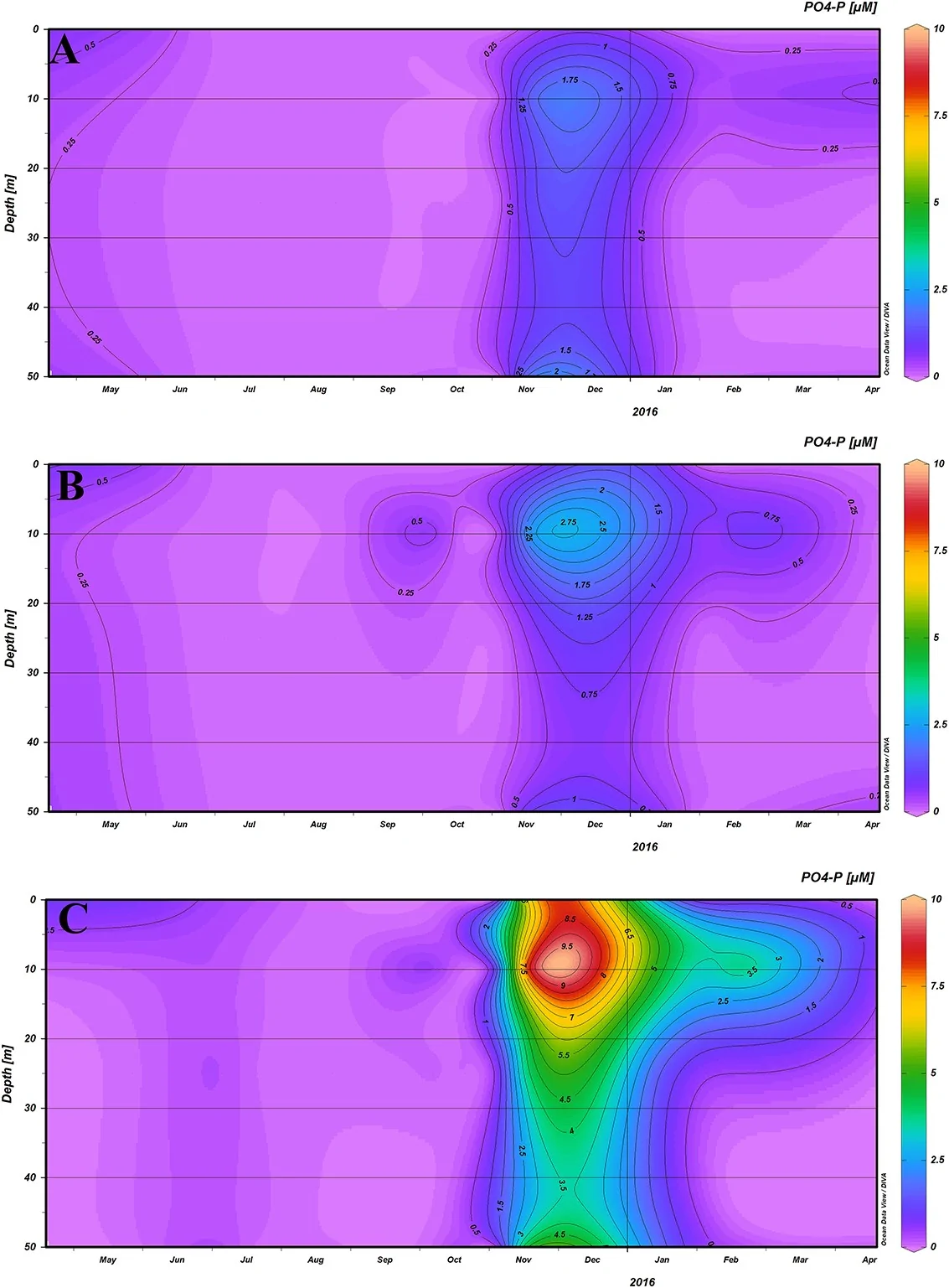
Spatio-temporal profiles of PO_4_ concentrations along the study area: (a) river mouth station, (b) coastal station, (c) offshore station.

Phosphate concentrations also exhibited pronounced spatial and vertical variability (Fig. 7). At the river mouth station, phosphate levels ranged between 0.001 μM (July) and 1.96 μM (December), while values at the coastal station varied between 0.001 μM (July) and 2.58 μM (December). At the offshore station, concentrations ranged from 0.003 μM (September) to 9.08 μM (December). Maximum phosphate concentrations were generally observed at ~10 m depth, and differences among sampling periods were statistically significant (ANOVA, *P* < 0.05). This vertical distribution indicates accumulation of phosphate below the surface layer.

**Fig. 8:**
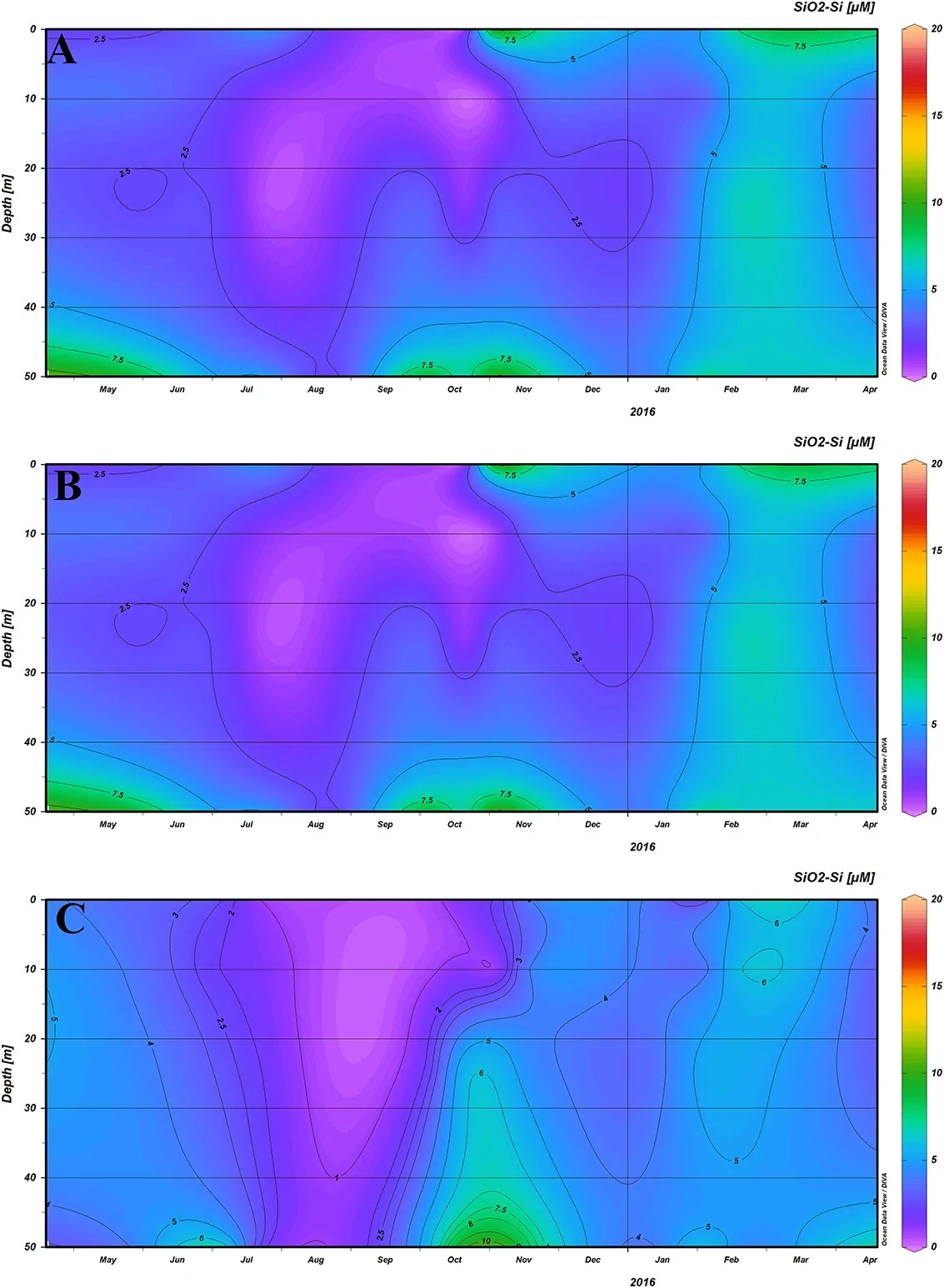
Spatio-temporal profiles of SiO_2_ concentrations along the study area: (a) river mouth station, (b) coastal station, (c) offshore station.

Silicate concentrations increased with depth across all stations (Fig. 8). Within the photic zone, silicate concentrations ranged between 0.04 μM (October) and 6.87 μM (November) at the river mouth station, 0.001 μM (October) and 16.45 μM (April) at the coastal station and 0.41 μM (November) and 10.72 μM (November) at the offshore station. Higher silicate concentrations were consistently observed in deeper layers and during winter months, and these differences were statistically significant (ANOVA, *P* < 0.05). The vertical gradient in silicate concentration was evident across all stations. Overall, nutrient distributions exhibited clear spatial and temporal patterns, with higher concentrations at river-influenced stations, lower values offshore and seasonal variability characterized by reduced concentrations in summer and elevated levels during autumn–winter periods.

### Distribution of diagnostic pigments

HPLC analysis revealed a diverse suite of diagnostic pigments representing major phytoplankton groups within the photic zone. Detected pigments included fucoxanthin, peridinin, zeaxanthin, chlorophyll-*b* and several accessory pigments, all of which exhibited marked variability in both vertical distribution and relative abundance.

Fucoxanthin, typically associated with diatoms and other chromophyte algae, showed higher concentrations in surface and upper photic layers, particularly at river mouth and coastal stations. Peridinin, a diagnostic pigment for dinoflagellates, was detected across multiple depths and stations, displaying considerable spatial variability. In contrast, zeaxanthin, commonly linked to cyanobacteria and picophytoplankton, tended to increase in relative importance toward deeper photic layers. These vertical differences indicate depth-dependent changes in phytoplankton community composition. Pigment concentrations varied substantially among stations and sampling periods (Supplementary Tables S1–S3). Chlorophyll-*a* concentrations were generally higher at the river mouth and coastal stations (Supplementary Table S2), whereas lower values were observed offshore. At nearshore stations, higher pigment concentrations were typically recorded in surface and subsurface layers, whereas offshore maxima were occasionally observed at deeper photic depths.

Among the accessory pigments, fucoxanthin, peridinin and 19′-hexanoyloxyfucoxanthin were the most prominent compounds following chlorophyll-*a*. Fucoxanthin concentrations ranged between 0.04–2.94 μg L^−1^ at the river mouth station, 0.04–3.34 μg L^−1^ at the coastal station and 0.03–2.61 μg L^−1^ at the offshore station, showing consistently higher values at nearshore stations. Peridinin concentrations ranged between 0.19–2.29 μg L^−1^, 0.14–2.64 μg L^−1^ and 0.11–1.76 μg L^−1^ at the river mouth, coastal and offshore stations, respectively, indicating its widespread occurrence across the study area. The third most abundant pigment, 19′-hexanoyloxyfucoxanthin, ranged between 0.04–0.98 μg L^−1^ at the river mouth station, 0.03–1.12 μg L^−1^ at the coastal station and 0.04–1.76 μg L^−1^ at the offshore station. Relatively higher concentrations at offshore stations indicate a shift in pigment composition along the coastal–offshore gradient. In contrast, zeaxanthin and chlorophyll-*b* were generally detected at lower concentrations, while 19′-butanoyloxyfucoxanthin and alloxanthin were occasionally below detection limits. Overall, the distribution of diagnostic pigments exhibited clear vertical and spatial variability, with higher concentrations of fucoxanthin and chlorophyll-*a* at nearshore stations and increased relative importance of small-cell-associated pigments toward offshore and deeper photic layers.

### Pigment-derived phytoplankton size structure

Estimates of phytoplankton size classes derived from diagnostic pigment ratios revealed pronounced vertical and spatial variability in community size structure across the photic zone. Microphytoplankton (>20 μm) constituted a substantial fraction of total phytoplankton biomass, particularly in the upper photic layers. Higher contributions of microphytoplankton were generally observed in surface waters, especially at river mouth and coastal stations. Nanophytoplankton (2–20 μm) represented a persistent and widely distributed component of the phytoplankton community, with relatively stable contributions across multiple depths. This size class was consistently present at all stations, showing intermediate contribution levels between micro- and picophytoplankton. In contrast, picophytoplankton (<2 μm) exhibited a distinct distribution pattern, with increasing relative contribution toward deeper photic layers and offshore stations. Higher picophytoplankton contributions were frequently observed in subsurface and lower photic depths.

**Fig. 9:**
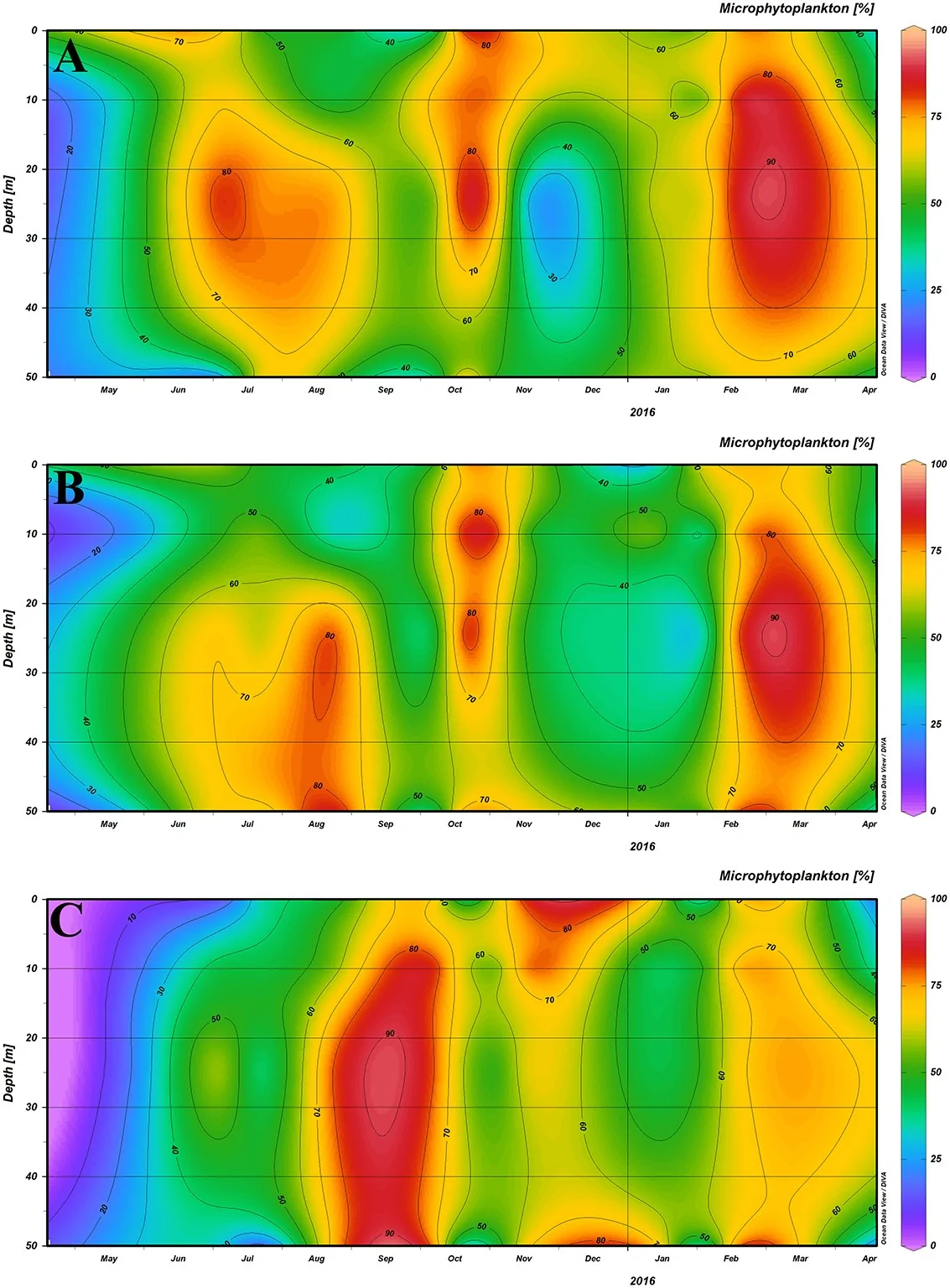
Relative contribution of microphytoplankton to total phytoplankton biomass at the river-mouth (a), coastal (b), and offshore (b) stations in the south-eastern Black Sea during monthly sampling. The figure shows that microphytoplankton generally dominated the upper photic zone, with the highest contributions at the river-mouth station and progressively lower contributions toward offshore waters.

**Fig. 10:**
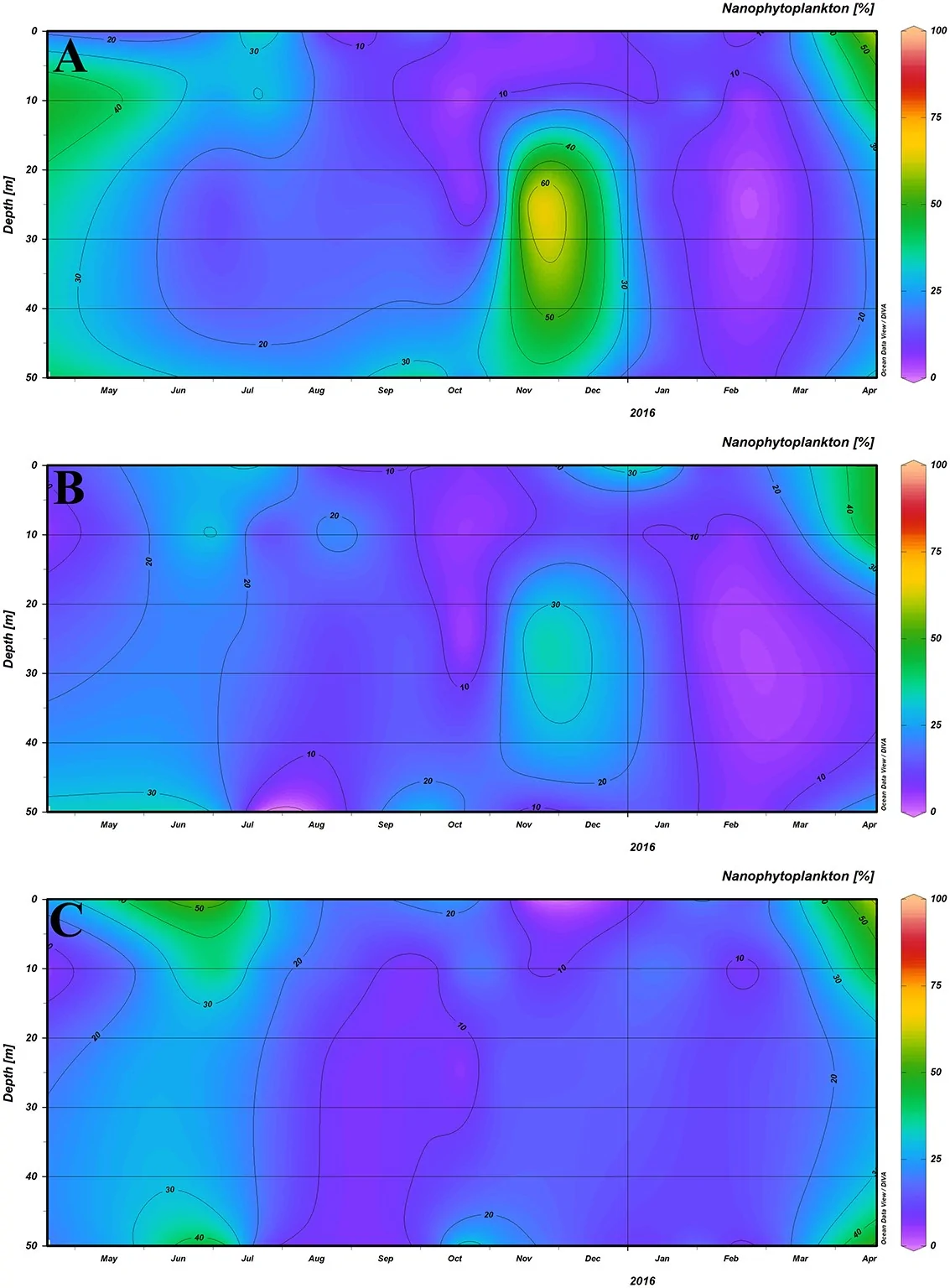
Spatio-temporal profiles of HPLC-derived nanophytoplankton along the study area: (a) river mouth station, (b) coastal station, (c) offshore station.

**Fig. 11:**
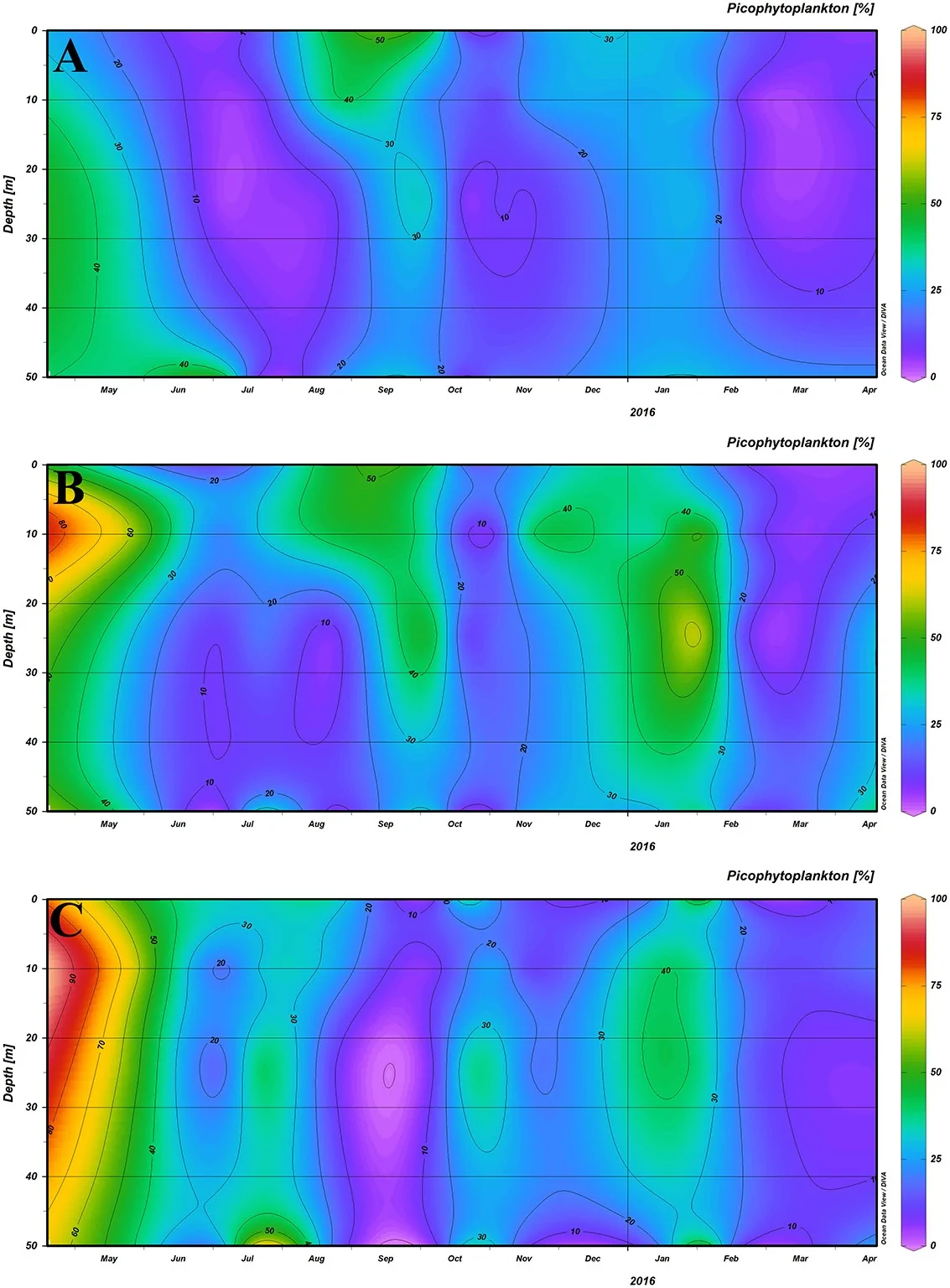
Spatio-temporal profiles of HPLC-derived picophytoplankton along the study area: (a) river mouth station, (b) coastal station, (c) offshore station.

Microphytoplankton was the dominant size class within the study area, with contribution rates ranging between 8% and 91% (Fig. 9). The highest contributions were recorded at the river mouth station, with a progressive decline toward coastal and offshore stations, indicating a clear coastal–offshore gradient. Vertical distribution patterns showed that microphytoplankton contributions were greatest within the upper 25 m of the water column. Nanophytoplankton contributions generally remained below 60% throughout the study period (Fig. 10). Higher contributions were observed at coastal and offshore stations compared to the river mouth station. In terms of vertical distribution, nanophytoplankton was present throughout the photic zone, with relatively elevated contributions in both surface and deeper photic layers. Picophytoplankton contributions ranged between 2% and 70%. The offshore station exhibited consistently higher picophytoplankton contributions (5–70%) compared to river mouth and coastal stations (Fig. 11). Vertical distribution analysis indicated that picophytoplankton contributions increased toward subsurface and lower photic depths, particularly at offshore and coastal stations. Overall, phytoplankton size structure exhibited clear spatial and vertical patterns, with microphytoplankton dominating in river-influenced surface waters, nanophytoplankton showing widespread distribution across all stations and picophytoplankton increasing in relative importance toward offshore and deeper photic environments.

### Vertical organization of phytoplankton communities

Overall, the results demonstrate that phytoplankton communities in the south-eastern Black Sea exhibit pronounced vertical structuring within the photic zone. Surface layers were generally characterized by higher phytoplankton biomass and a greater contribution of larger phytoplankton size classes, whereas deeper layers showed increasing relative contributions of smaller phytoplankton groups. This pattern indicates a clear depth-dependent organization of phytoplankton size structure. Microphytoplankton contributions were predominantly higher in surface and upper photic layers, particularly at river mouth and coastal stations, while nano- and picophytoplankton contributions increased toward subsurface and deeper photic depths. This vertical distribution pattern was consistently observed across stations and sampling periods.

The observed vertical variability corresponds to gradients in environmental conditions within the water column, including changes in light availability and hydrographic structure. In addition, the co-occurrence of surface biomass maxima and subsurface chlorophyll maxima indicates a vertically structured phytoplankton distribution within the photic zone. Overall, the combined results from hydrographic measurements, chlorophyll-*a* distribution, nutrient concentrations and pigment-derived size classes demonstrate a consistent vertical and spatial organization of phytoplankton communities across the study area.

### Environmental controls on phytoplankton size structure (CCA)

**Fig. 12:**
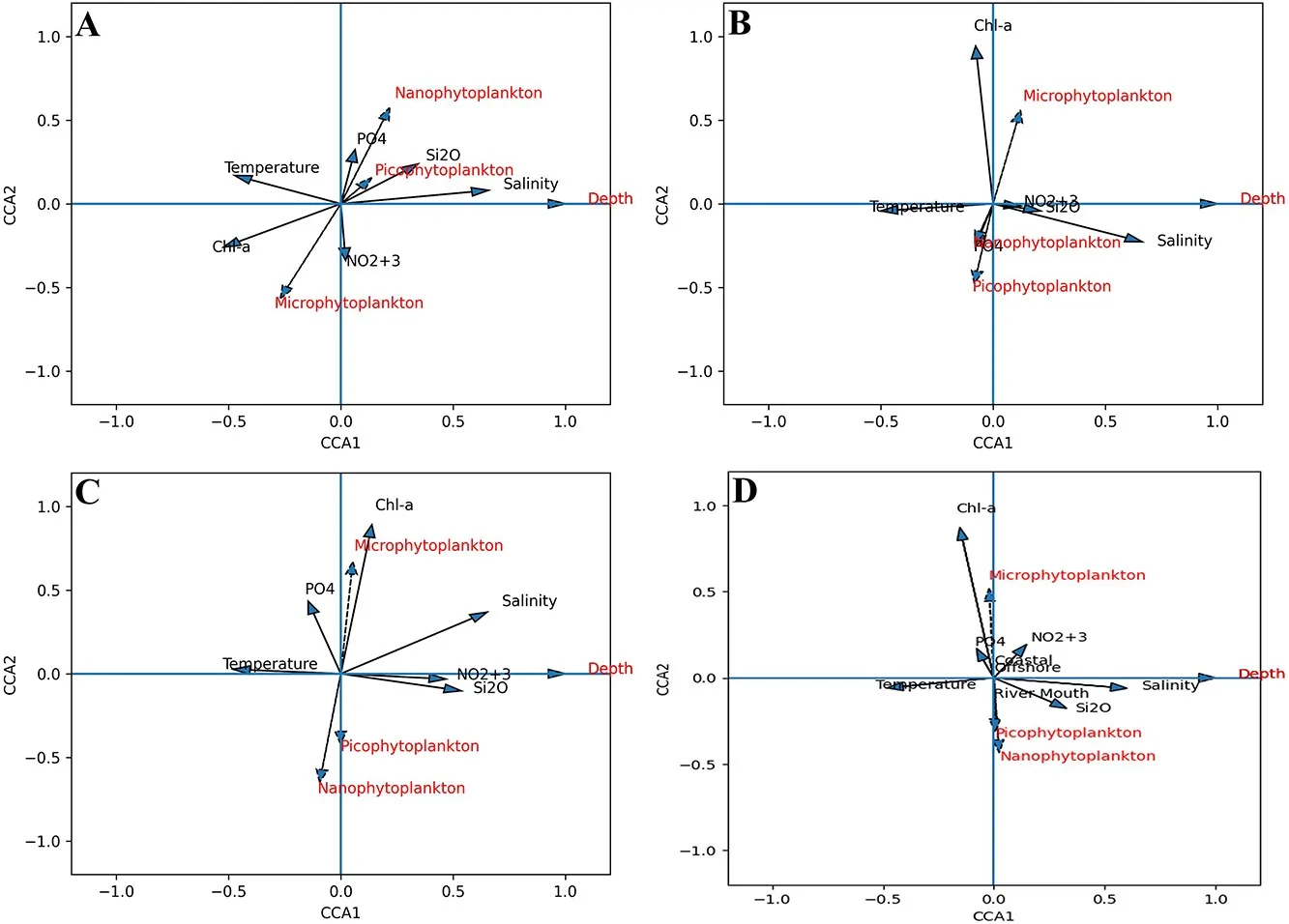
Canonical Correspondence Analysis (CCA) ordination diagrams illustrating the relationships between phytoplankton size classes and environmental variables in the south-eastern Black Sea. (a) river mouth station, (b) coastal station, (c) offshore station and (d) combined ordination including all sampling stations. Blue arrows represent environmental variables, whereas red arrows indicate phytoplankton size classes. The relative orientation and length of the vectors illustrate the strength and direction of the relationships between environmental gradients and phytoplankton size structure.

To identify the primary environmental drivers shaping phytoplankton size structure across the study area, a CCA was performed by integrating size–class composition with hydrographic and nutrient variables (Fig. 12a–d). The CCA ordination revealed a clear separation of samples along the first canonical axis (CCA1), representing a strong cross-shelf gradient associated primarily with nutrient availability and salinity. The first two canonical axes had eigenvalues of 0.0003 and 0.00006, respectively, with CCA1 explaining 84.33% and CCA2 15.67% of the constrained variation represented by the first two axes. Permutation-based Monte Carlo tests confirmed that the overall CCA model was statistically significant (pseudo-*F* = 0.103, *P* = 0.001, 999 permutations), with both the first (*P* = 0.001) and second canonical axes (*P* = 0.008) contributing significantly to the observed phytoplankton–environment relationships. These results indicate that the primary variability in phytoplankton size structure is aligned with the nutrient–salinity gradient, while secondary variability is associated with temperature-related processes.

Station-specific analyses revealed distinct environmental controls at each sampling location. At the river mouth station, the first two canonical axes explained 82.44% and 17.56% of the constrained variation, respectively. The overall CCA model was significant (pseudo-*F* = 0.154, *P* = 0.005). Microphytoplankton was strongly associated with elevated chlorophyll-*a* concentrations and nutrient-rich conditions, whereas nanophytoplankton and picophytoplankton showed positive relationships with salinity, phosphate and silicate concentrations. Temperature exhibited an opposite orientation to salinity and nutrient-related variables, indicating contrasting seasonal environmental conditions (Fig. 12a). At the coastal station, CCA1 and CCA2 explained 75.30% and 24.70% of the constrained variation, respectively. The ordination was also statistically significant (pseudo-*F* = 0.128, *P* = 0.022). Microphytoplankton was positively associated with chlorophyll-*a*, while nanophytoplankton and picophytoplankton were more closely related to salinity and nutrient gradients. Temperature was positioned opposite to salinity along the primary axis, reflecting the influence of seasonal hydrographic variability on phytoplankton size structure (Fig. 12b). At the offshore station, CCA1 accounted for 97.50% of the variation and represented a strong environmental gradient associated primarily with salinity, depth, nitrate and silicate concentrations. The CCA model was highly significant (pseudo-*F* = 0.169, *P* = 0.002). Microphytoplankton was positively related to chlorophyll-*a* and phosphate, whereas pico- and nanophytoplankton were associated with deeper and more saline waters. The dominance of CCA1 indicates that offshore phytoplankton size structure was largely controlled by a single environmental gradient (Fig. 12c).

The combined CCA ordination (Fig. 12d) showed a clear environmental transition from nutrient-enriched river mouth conditions to more saline offshore waters. River mouth samples were associated with higher nutrient availability and chlorophyll-*a* concentrations, whereas offshore samples were related to salinity, depth and silicate enrichment. Coastal samples occupied an intermediate position between these two endmembers, reflecting transitional environmental conditions along the cross-shelf gradient.

Phytoplankton size classes exhibited distinct responses to environmental forcing. Microphytoplankton was closely associated with elevated chlorophyll-*a* concentrations and nutrient-enriched conditions, while picophytoplankton and nanophytoplankton were generally linked to more saline and deeper-water environments. These relationships were consistent with the Pearson correlation analysis (Supplementary Table S4), which showed significant positive correlations between chlorophyll-*a* and microphytoplankton, whereas smaller phytoplankton size classes were more closely associated with increasing salinity and reduced nutrient availability. These patterns indicate a progressive shift from larger phytoplankton forms in productive coastal and river-influenced waters toward smaller size classes under increasingly oligotrophic offshore conditions. Detailed statistical outputs supporting the CCA and correlation analyses are presented in Supplementary Table S5.

Overall, the CCA results demonstrate that salinity, nutrient availability, chlorophyll-*a* concentration and depth were the principal environmental variables structuring phytoplankton size composition in the south-eastern Black Sea. The consistently high proportion of variation explained by the first canonical axis across all station-specific and combined analyses emphasizes the dominant role of cross-shelf environmental gradients in regulating phytoplankton community size structure. Although the absolute eigenvalues were numerically small, this is common for species-composition datasets analyzed using CCA, and the statistically significant permutation tests indicate that the identified environmental gradients explain a meaningful and non-random component of phytoplankton size–class variability. The agreement between station-specific and combined ordinations further supports the robustness of the observed phytoplankton–environment relationships.

## DISCUSSION

### Hydrographic forcing and seasonal structuring of the photic zone

The hydrographic structure of the Black Sea plays a fundamental role in regulating phytoplankton community dynamics. As a semi-enclosed basin characterized by strong vertical stratification, the Black Sea exhibits pronounced seasonal variability in the upper water column. The present results demonstrate that phytoplankton size structure in the south-eastern Black Sea is tightly coupled to this seasonal hydrographic variability, linking physical forcing directly to ecological organization within the photic zone. Surface temperatures ranged between ~9.7°C in winter and 28.2°C in summer, while salinity increased with depth from ~16–18‰ at the surface to ~19–20‰ below the upper mixed layer. Such stratification patterns are consistent with previous observations in the Black Sea and represent a key mechanism controlling nutrient transport and biological productivity (Oguz *et al.,* 2003; Oguz and Velikova, 2010).

The seasonal development of the thermocline restricts vertical nutrient fluxes from deeper waters and promotes the formation of a stable photic layer. Under these conditions, phytoplankton communities reorganize according to physiological traits that enable efficient resource acquisition under stratified conditions (Margalef, 1978; Cullen, 2015). In this context, the observed increase in nano- and picophytoplankton contributions during stratified periods indicates that smaller cells gain a competitive advantage under nutrient-depleted conditions, consistent with size-dependent resource utilization theory. Conversely, the breakdown of stratification during winter enhances nutrient availability in surface waters and favours larger phytoplankton taxa capable of rapid growth under nutrient-replete conditions (Falkowski and Raven, 2007; Marañón, 2015). The dominance of microphytoplankton during these periods therefore supports the hypothesis that mixing-driven nutrient enrichment promotes larger phytoplankton, whereas stratification favors smaller size classes.

Seasonal temperature variations observed in the present study were consistent with the typical thermal cycle of the Black Sea, where sea surface temperatures increase during spring, reach maximum values in summer and decline during winter mixing (Ivanov, 1985). The recorded temperature range (9.7–28.2°C) is comparable to previous regional studies (Sivri, 1999; Agirbas, 2010; Kopuz, 2012; Koca, 2014; Bakırcı, 2017). The relatively elevated summer temperatures observed during the study period may reflect interannual variability or ongoing regional warming, which can intensify stratification and further limit nutrient supply to surface waters. Such warming-induced stratification has been widely associated with shifts toward smaller phytoplankton communities in marine ecosystems (Behrenfeld *et al.,* 2006; Doney *et al.,* 2012).

Salinity profiles also exhibited the characteristic vertical structure of the Black Sea, with lower salinity surface waters (16–18‰) overlying more saline deeper waters (19–21‰). This haline stratification reinforces vertical stability and further constrains nutrient exchange between surface and deeper layers. Consequently, phytoplankton productivity is strongly influenced by episodic mixing events and localized nutrient inputs. The elevated phytoplankton biomass and increased contribution of microphytoplankton at the river mouth station indicate that coastal nutrient enrichment enhances the dominance of larger phytoplankton groups, supporting the hypothesis that spatial gradients in nutrient availability regulate phytoplankton size structure. Overall, the hydrographic forcing identified in this study acts as a primary control on phytoplankton size structure, regulating both seasonal and spatial variability through its influence on nutrient availability, stratification and water column stability.

### Photic zone structure and subsurface chlorophyll maxima

The photic depth in the study area ranged between ~15 and 38 m depending on station and season, reflecting variability in water transparency and suspended particle loads. This variability indicates that light attenuation in the south-eastern Black Sea is controlled by both phytoplankton biomass and terrigenous inputs, particularly at river-influenced and coastal stations. Chlorophyll-*a* concentrations frequently exhibited subsurface maxima near the mid-photic zone or around the 10% PAR level. The recurrent formation of subsurface chlorophyll maxima (SCM) highlights the presence of a vertically structured photic environment, where optimal growth conditions are not restricted to the surface layer. The occurrence of SCM is a well-established feature of stratified marine systems and reflects a balance between light availability and nutrient supply (Fennel and Boss, 2003).

In stratified systems, surface waters often become nutrient depleted due to biological uptake and reduced vertical exchange. Under these conditions, phytoplankton biomass is redistributed toward deeper photic layers, where sufficient light and relatively higher nutrient availability coexist. The nutrient profiles observed in this study, particularly subsurface phosphate enrichment and increasing silicate concentrations with depth, support this vertical redistribution of biomass. Similar SCM structures have been reported in other regions of the Black Sea during stratified periods (Yunev *et al.,* 2002; Oguz and Velikova, 2010). The consistent presence of SCM layers in this study therefore indicates that the photic zone functions as a vertically heterogeneous habitat rather than a uniform production layer. The formation of SCM layers is closely linked to phytoplankton size structure. The observed increase in nano- and picophytoplankton contributions at deeper photic depths suggests that smaller phytoplankton are favored under stratified and resource-limited conditions, supporting the hypothesis that size-dependent ecological strategies govern phytoplankton distribution. Furthermore, the co-occurrence of SCM formation and reduced surface nutrient availability indicates that stratification promotes vertical niche differentiation within the phytoplankton community. Although the euphotic depth occasionally occurred above 50 m, the inclusion of 50 m samples enabled characterization of the lower photic–sub-euphotic transition, where residual pigment biomass and nutrient enrichment may persist despite irradiance levels below the conventional 1% PAR threshold. Retaining these samples therefore provided a more comprehensive representation of vertical phytoplankton distribution and the environmental gradients influencing community structure. Overall, these results demonstrate that photic zone structure, and particularly SCM dynamics, act as a key regulator of phytoplankton distribution by linking light availability, nutrient gradients and size-dependent community responses in the south-eastern Black Sea.

### Nutrient gradients and coastal–offshore contrasts

Nutrient distributions further emphasize the importance of environmental gradients in shaping phytoplankton communities. Nitrite + nitrate concentrations remained generally below 5 μM but were consistently higher at the river-mouth station, reflecting the influence of terrestrial nutrient inputs. Phosphate concentrations showed pronounced variability and were frequently elevated in subsurface layers, whereas silicate concentrations increased with depth and during periods of vertical mixing. These patterns are consistent with the hydrographic characteristics of the Black Sea, where nutrient dynamics are regulated by the combined effects of river discharge, internal recycling and seasonal mixing processes (Coban-Yildiz *et al.,* 2000; Oguz *et al.,* 2003; Agirbas *et al*., 2015).

The spatial distribution of nutrients defines a clear coastal–offshore gradient, with nutrient-rich nearshore waters transitioning toward more oligotrophic offshore conditions. This gradient provides a fundamental framework controlling both phytoplankton biomass and size structure. Coastal waters receiving riverine inputs sustain higher phytoplankton biomass, whereas offshore regions are characterized by stronger stratification and reduced nutrient availability (Yunev *et al.,* 2002; Moncheva *et al.,* 2012). The observed decline in chlorophyll-*a* concentrations from river-influenced stations toward offshore waters directly reflects this gradient. In addition, the dominance of microphytoplankton in nutrient-enriched coastal environments and the increasing importance of nano- and picophytoplankton offshore indicate that spatial differences in nutrient availability regulate phytoplankton size composition, supporting the hypothesis that coastal–offshore gradients structure community organization.

Nutrient availability represents a primary control on phytoplankton growth in marine ecosystems, and in the Black Sea, it is strongly influenced by both external inputs and internal biogeochemical cycling. Nitrite + nitrate concentrations exhibited clear seasonal variability, reflecting dynamic nutrient cycling within the photic zone. The measured concentrations (0.001–2.12 μM) fall within the range reported for the southern Black Sea, although they are generally lower than some previous observations (Coban-Yildiz *et al.,* 2000; Agirbas, 2010). These relatively low concentrations indicate that nutrient limitation, particularly during stratified periods, is a dominant feature of the study area.

Phosphate concentrations in the present study were comparatively low relative to historical records from other regions of the Black Sea. Previous studies have shown that phosphate levels are strongly influenced by terrestrial inputs and biological uptake (Brewer and Murray, 1973; Bologa, 1986; Zaitsev, 1992). While average surface phosphate concentrations in the Black Sea have been reported at ~0.42 μM (Sorokin, 1986), values in the north-western basin may reach up to 6.39 μM (Bologa, 1986). The comparatively lower phosphate concentrations observed in this study suggest a shift toward more oligotrophic conditions in the south-eastern Black Sea. An exceptionally high phosphate concentration (9.08 μM) was recorded in a single offshore sample during December. Although this value exceeds typical phosphate concentrations reported for offshore photic waters of the Black Sea, verification of the original laboratory records and analytical dataset confirmed the validity of the measurement. The elevated phosphate concentration was observed during the winter mixing period (December), when nutrient enrichment of the upper water column was also reflected by increased nitrate and silicate concentrations. This isolated observation may reflect localized nutrient enrichment, short-term hydrographic variability or episodic mixing processes and did not influence the overall spatial and seasonal nutrient patterns observed during the study period. Furthermore, the subsurface enrichment of phosphate provides a mechanistic explanation for the development of subsurface chlorophyll maxima and the vertical redistribution of phytoplankton biomass.

Silicate concentrations exhibited clear seasonal and vertical variability, with higher values observed during periods of increased vertical mixing. Since silicate is essential for diatom growth, fluctuations in its availability can strongly influence phytoplankton composition. The observed range (0.01–13.63 μM) is consistent with previous studies in the south-eastern Black Sea (Ağırbaş, 2010; Kopuz, 2012; Koca, 2014). Elevated silicate concentrations during mixing periods favor the dominance of microphytoplankton, whereas reduced availability under stratified conditions is associated with a shift toward smaller phytoplankton size classes, supporting the hypothesis that seasonal nutrient dynamics regulate size structure.

Overall, the nutrient patterns identified in this study demonstrate a strong coupling between hydrographic forcing, nutrient availability and phytoplankton size structure, with coastal enrichment and offshore nutrient limitation driving spatial variability, and seasonal depletion–replenishment cycles regulating temporal changes in community composition.

### Diagnostic pigments and taxonomic signals

Phytoplankton community structure in marine ecosystems is closely linked to environmental conditions, particularly nutrient availability and water column stratification. Diagnostic pigment analysis, enabled by HPLC techniques, provides a powerful tool for resolving functional and taxonomic variability that cannot be fully captured by microscopy-based approaches (Mantoura and Llewellyn, 1983; Mackey *et al.,* 1996; Jeffrey and Vesk, 1997). In the present study, fucoxanthin, peridinin and 19′-hexanoyloxyfucoxanthin were identified as the dominant marker pigments, indicating that diatoms, dinoflagellates and haptophytes constitute major components of the phytoplankton community in the south-eastern Black Sea. Pigment concentrations were generally higher than those reported in earlier regional studies, particularly at river-influenced stations, suggesting that localized nutrient enrichment enhances both biomass and taxonomic diversity.

The high abundance of fucoxanthin indicates a strong contribution of diatom-dominated assemblages, particularly in nearshore and nutrient-enriched environments. Diatoms are known to respond rapidly to nutrient inputs and mixing events and frequently dominate productive coastal systems (Falkowski and Raven, 2007). The spatial distribution of fucoxanthin therefore reflects a direct coupling between nutrient availability and microphytoplankton dominance. The presence of peridinin indicates the contribution of dinoflagellates, which are often associated with stratified conditions and exhibit adaptive strategies such as vertical migration and mixotrophy (Smayda and Reynolds, 2001). The widespread occurrence of peridinin across depths and stations suggests that dinoflagellates contribute to community persistence under variable environmental conditions. The detection of 19′-hexanoyloxyfucoxanthin indicates the presence of haptophytes, including coccolithophores, which are common in Black Sea phytoplankton assemblages (Moncheva *et al.,* 2012). These groups are typically associated with moderately stratified and nutrient-limited conditions and may contribute to carbon export through calcification processes (Paasche, 2001). The relatively higher contribution of this pigment in offshore waters indicates an increasing importance of nanophytoplankton under nutrient-depleted conditions, consistent with a shift toward smaller size classes under stratification.

Chlorophyll-*a* concentrations observed in this study were slightly higher than some historical records from the Black Sea. Long-term observations indicate surface chlorophyll-*a* concentrations of ~0.15 ± 0.04 μg L^−1^ during 1964–1986 (Yunev *et al.,* 2002), while later studies reported basin-wide averages between 0.59 and 0.69 μg L^−1^ (Kopelevich *et al.,* 2002). In the present study, surface chlorophyll-*a* ranged between 0.51–3.97 μg L^−1^ at the river mouth station, 0.16–2.47 μg L^−1^ at the coastal station and 0.18–3.04 μg L^−1^ at the offshore station. These elevated values, particularly in coastal areas, likely reflect enhanced nutrient inputs and regional variability while also highlighting the importance of methodological consistency when comparing pigment-based datasets across studies. Importantly, despite the increasing contribution of nano- and picophytoplankton under stratified conditions, pigment composition indicates that eukaryotic phytoplankton groups (diatoms, dinoflagellates and haptophytes) remain dominant. Although zeaxanthin is widely used as a diagnostic pigment for cyanobacteria, it may also be present in prochlorophytes and, to a lesser extent, certain picoeukaryotes under oligotrophic conditions. Consequently, the picophytoplankton fractions estimated using the diagnostic pigment approach should be interpreted as representing the combined contribution of zeaxanthin-containing picophytoplankton rather than cyanobacteria alone. Nevertheless, previous studies indicate that cyanobacteria constitute the dominant zeaxanthin-bearing picophytoplankton group in the Black Sea (Yunev *et al.,* 2002; Moncheva *et al.,* 2012), supporting the applicability of the Vidussi *et al.* (2001) approach while acknowledging a limited degree of taxonomic uncertainty. Future studies integrating HPLC pigment analysis with molecular or flow-cytometric techniques would further improve the taxonomic resolution of picophytoplankton communities in the south-eastern Black Sea. Although the diagnostic pigment approach proposed by Vidussi *et al.* (2001) has been widely used to estimate phytoplankton size structure in marine ecosystems, the weighting coefficients were originally derived from open-ocean phytoplankton assemblages. Consequently, their application to semi-enclosed basins such as the Black Sea should be interpreted with some caution, as regional differences in species composition, pigment expression and environmental conditions may influence the quantitative allocation of phytoplankton size classes. Nevertheless, the method has been successfully applied in numerous coastal and marginal marine environments and remains a robust and widely accepted framework for comparative assessments of phytoplankton functional structure. Accordingly, the phytoplankton size–class estimates presented in this study should be interpreted as ecologically meaningful indicators of functional community structure rather than absolute taxonomic abundances. This suggests that the south-eastern Black Sea does not exhibit a complete shift toward picoplankton-dominated oligotrophic conditions but rather a dynamic and transitional community structure shaped by interacting environmental drivers.

Overall, the diagnostic pigment signatures demonstrate that phytoplankton community composition is strongly regulated by nutrient availability and stratification, with diatoms dominating under nutrient-enriched conditions and smaller phytoplankton groups increasing in relative importance under stratified, nutrient-limited environments, supporting the hypothesis that environmental gradients drive both taxonomic composition and size structure in the south-eastern Black Sea.

### Phytoplankton size structure and ecosystem implications

The size structure of phytoplankton communities provides critical insights into trophic dynamics and ecosystem functioning in marine environments. In general, microphytoplankton dominate under nutrient-rich conditions, whereas picophytoplankton become more prevalent in oligotrophic systems (Zubkov *et al.,* 1998; Gibb *et al.,* 2000; Barlow *et al.,* 2002). The present study shows that microphytoplankton constituted the dominant size class, contributing between 8% and 91% of total phytoplankton biomass. This dominance was most pronounced in coastal and river-influenced waters, where nutrient inputs and mixing processes support the growth of larger phytoplankton taxa. These findings indicate that coastal regions of the south-eastern Black Sea maintain relatively high productivity levels capable of sustaining fast-growing microphytoplankton assemblages.

In contrast, nano- and picophytoplankton exhibited higher relative contributions in offshore waters and during stratified periods. The increased importance of picophytoplankton under these conditions reflects their physiological advantages, including high nutrient uptake efficiency and adaptability to low-nutrient environments. This offshore shift toward smaller size classes indicates a transition toward more oligotrophic conditions away from coastal influence and supports the hypothesis that nutrient limitation and stratification promote the dominance of smaller phytoplankton groups. Nanophytoplankton displayed a more complex distribution pattern, with elevated contributions near the deep chlorophyll maximum and the lower boundary of the photic zone. This distribution suggests that nanophytoplankton occupy an intermediate ecological niche, responding to both light availability and nutrient gradients. The co-occurrence of nanophytoplankton maxima with SCM layers indicates that vertical niche differentiation and resource partitioning play a key role in structuring phytoplankton communities.

Phytoplankton cell size is a key ecological trait influencing nutrient uptake, growth rate and trophic transfer efficiency (Chisholm, 1992; Marañón, 2015). Larger phytoplankton cells, such as diatoms, are typically favored under nutrient-rich conditions, whereas smaller cells are more competitive under nutrient limitation due to their higher surface-area-to-volume ratios (Kiørboe, 1993). The coexistence of microphytoplankton dominance in coastal waters and increasing nano- and picophytoplankton contributions offshore indicates that the south-eastern Black Sea functions as a transitional system where eutrophic and oligotrophic processes overlap. This pattern is consistent with the spatial organization revealed by CCA, where nutrient-rich, low-salinity environments are associated with microphytoplankton, while high-salinity, nutrient-limited offshore conditions favor smaller size classes.

From an ecosystem perspective, these size-structured shifts have important implications for trophic transfer efficiency and biogeochemical cycling. Microphytoplankton-dominated systems generally support more efficient energy transfer to higher trophic levels, including commercially important fish species, whereas picophytoplankton-dominated systems tend to enhance microbial loop pathways and reduce trophic efficiency. Therefore, the observed increase in smaller phytoplankton size classes under stratified and offshore conditions suggests a potential shift in energy flow pathways and carbon cycling within the ecosystem. Overall, these results demonstrate that phytoplankton size structure in the south-eastern Black Sea is primarily regulated by the interaction between nutrient availability, stratification and spatial gradients, confirming that environmental forcing drives both community composition and ecosystem functioning, with important implications for regional productivity and food web dynamics.

### Ecological implications for the Black Sea ecosystem

The spatial variability in phytoplankton size structure has important implications for ecosystem functioning in the south-eastern Black Sea. Larger phytoplankton cells generally support more efficient trophic transfer to mesozooplankton and fish populations, whereas smaller cells tend to channel primary production through microbial food webs (Legendre and Rassoulzadegan, 1995). Accordingly, microphytoplankton-dominated systems enhance carbon transfer to higher trophic levels, while pico- and nanophytoplankton dominance promotes recycling within the microbial loop. In the present study, the clear spatial and vertical gradients in phytoplankton size structure indicate that these contrasting trophic pathways coexist within the south-eastern Black Sea, resulting in a highly heterogeneous and spatially structured ecosystem.

The coexistence of distinct size-structured regimes suggests that the pelagic ecosystem operates as a mosaic of trophic pathways regulated by environmental gradients. Nutrient-enriched coastal regions, influenced by riverine inputs and episodic mixing, are characterized by microphytoplankton dominance and are likely associated with higher trophic transfer efficiency and enhanced fisheries productivity. In contrast, offshore and stratified environments are increasingly dominated by nano- and picophytoplankton, indicating a shift toward microbial loop–dominated pathways and reduced energy transfer efficiency to higher trophic levels.

This spatial decoupling of trophic pathways has important implications for carbon cycling. Microphytoplankton contribute more effectively to carbon export via sinking processes, whereas smaller phytoplankton primarily support regenerated production within the upper water column. Furthermore, the observed increase in smaller phytoplankton size classes under stratified conditions suggests a potential transition toward reduced trophic efficiency in the Black Sea ecosystem. Such changes are consistent with global observations showing that warming and enhanced stratification favor smaller phytoplankton communities, with cascading effects on food web structure and biogeochemical cycling (Behrenfeld *et al.,* 2006; Doney *et al.,* 2012).

Importantly, the coexistence of productive (microphytoplankton-dominated) and recycling-dominated (pico/nano-dominated) regimes indicates that the south-eastern Black Sea currently functions as a transitional ecosystem, where localized nutrient inputs partially offset broader oligotrophication trends. Overall, these findings demonstrate that phytoplankton size structure is a key ecological indicator linking environmental gradients to trophic dynamics, carbon cycling and ecosystem resilience, highlighting the sensitivity of the Black Sea to ongoing climate-driven change.

### Methodological implications of pigment-based approaches

The present study highlights the value of pigment-based approaches for assessing phytoplankton community structure in marine ecosystems. HPLC enables rapid, reproducible and high-resolution quantification of phytoplankton functional groups and size classes and has become a widely adopted tool in marine ecological research (Mackey *et al.,* 1996; Jeffrey and Vesk, 1997). Its capacity to resolve spatial and temporal variability makes it particularly well suited for dynamic and heterogeneous systems such as the Black Sea.

Despite these advantages, pigment-based approaches have inherent limitations. HPLC analysis does not provide species-level taxonomic resolution, and overlapping pigment signatures among phytoplankton groups may introduce uncertainty in taxonomic attribution. In addition, physiological variability driven by environmental factors such as light intensity, nutrient availability and growth conditions can influence pigment composition within individual taxa, further complicating interpretation. Consequently, pigment-based estimates of phytoplankton size structure should be interpreted with caution, particularly when applied as standalone indicators.

Nevertheless, the strong agreement between pigment-derived size structure and the observed hydrographic and nutrient gradients in this study supports the reliability of this approach for capturing large-scale ecological patterns. This consistency indicates that pigment-based methods are effective for resolving size-structured responses to environmental forcing, even in complex coastal–offshore systems. To further improve taxonomic resolution and reduce uncertainty, future studies should integrate pigment analysis with complementary techniques such as microscopy, flow cytometry and molecular approaches (e.g. metabarcoding). Such multi-method frameworks would enable cross-validation of results and provide a more comprehensive understanding of phytoplankton community dynamics across scales. Overall, HPLC-based pigment analysis represents a robust and efficient approach for monitoring phytoplankton community structure and detecting ecosystem-scale changes, particularly when combined with complementary analytical methods.

Overall, the findings support the hypotheses proposed in this study by demonstrating that phytoplankton size structure varies predictably along the river–coastal–offshore gradient in response to environmental forcing. The observed spatial organization of phytoplankton communities, together with the CCA results, confirms that hydrographic structure, nutrient availability, salinity and water-column stratification act as the principal environmental drivers regulating phytoplankton size composition in the south-eastern Black Sea. These findings further demonstrate that pigment-based approaches, when interpreted alongside environmental observations, provide a reliable framework for investigating phytoplankton functional structure and ecosystem responses to environmental variability.

## CONCLUSION

This study provides a comprehensive assessment of the vertical and spatial variability of pigment-derived phytoplankton size structure throughout the photic zone of the south-eastern Black Sea. The results demonstrate that phytoplankton community organization is strongly regulated by the interaction between hydrographic structure, nutrient availability and light availability, with seasonal stratification and cross-shelf environmental gradients representing the primary mechanisms controlling size-dependent community composition.

The development of a stratified photic layer promoted the formation of subsurface chlorophyll maxima, highlighting the importance of vertical environmental heterogeneity in structuring phytoplankton communities. Pigment analysis revealed that diatoms and dinoflagellates remained the dominant phytoplankton groups, while haptophytes and smaller phytoplankton size classes increased in relative importance under stratified and nutrient-limited conditions. Microphytoplankton predominated in nutrient-enriched river-influenced and coastal waters, whereas nano- and picophytoplankton became progressively more important toward offshore oligotrophic environments.

The coexistence of productive coastal communities dominated by microphytoplankton and offshore assemblages characterized by increasing contributions of nano- and picophytoplankton demonstrates that the south-eastern Black Sea functions as a transitional ecosystem linking eutrophic and oligotrophic processes across spatial and vertical gradients. Together, these findings demonstrate that phytoplankton size structure provides a sensitive ecological indicator linking environmental variability with ecosystem functioning. The strong explanatory power of the first canonical axis across all CCA analyses further emphasizes that cross-shelf environmental gradients represent the dominant mechanism structuring phytoplankton communities in the south-eastern Black Sea.

From an ecosystem perspective, the observed shift toward smaller phytoplankton under increasingly stratified conditions suggests potential changes in trophic transfer efficiency and carbon cycling, with greater reliance on microbial pathways under future climate-driven environmental change. Accordingly, long-term integrated monitoring programmes combining pigment analysis with complementary approaches, including microscopy, flow cytometry and molecular techniques, will be essential for improving predictions of phytoplankton dynamics and ecosystem responses in the Black Sea.

## Supplementary Material

Supplementary_materials_fbag063

## Data Availability

The data supporting the findings of this study are available from the corresponding author upon reasonable request.
