# Supplementary material for: Photic zone dynamics and environmental controls of pigment-derived phytoplankton size classes in the South-Eastern Black Sea
Source: J Plankton Res. 2026 Aug 21;48(5):fbag063. doi: 10.1093/plankt/fbag063 (PMC13496243; doi:10.1093/plankt/fbag063)
Supplement: Supplementary file 1 [file supplementary_materials_fbag063.zip › Supplementary_materials_fbag063_Table 6.docx]

**Supplementary Table S6.** Tukey HSD pairwise comparisons among stations (Tukey HSD post hoc comparisons following one-way ANOVA. Significant = adjusted p < 0.05.)

| **Variable** | **Comparison** | **Mean difference** | **95% CI** | **Adjusted p** | **Significant** |
| --- | --- | --- | --- | --- | --- |
| Microphytoplankton (%) | Coastal vs Offshore | -4.130 | -13.707 to 5.448 | 0.565 | No |
| Microphytoplankton (%) | Coastal vs River Mouth | 2.598 | -6.980 to 12.175 | 0.797 | No |
| Microphytoplankton (%) | Offshore vs River Mouth | 6.727 | -2.850 to 16.305 | 0.223 | No |
| Nanophytoplankton (%) | Coastal vs Offshore | 4.519 | -1.614 to 10.652 | 0.192 | No |
| Nanophytoplankton (%) | Coastal vs River Mouth | 3.300 | -2.832 to 9.434 | 0.412 | No |
| Nanophytoplankton (%) | Offshore vs River Mouth | -1.219 | -7.352 to 4.914 | 0.885 | No |
| Picophytoplankton (%) | Coastal vs Offshore | -0.390 | -7.709 to 6.930 | 0.991 | No |
| Picophytoplankton (%) | Coastal vs River Mouth | -5.898 | -13.217 to 1.421 | 0.140 | No |
| Picophytoplankton (%) | Offshore vs River Mouth | -5.508 | -12.828 to 1.811 | 0.179 | No |
| NO₂+NO₃ (µM) | Coastal vs Offshore | -0.037 | -0.368 to 0.294 | 0.963 | No |
| NO₂+NO₃ (µM) | Coastal vs River Mouth | 0.213 | -0.118 to 0.544 | 0.282 | No |
| NO₂+NO₃ (µM) | Offshore vs River Mouth | 0.250 | -0.081 to 0.581 | 0.178 | No |
| PO₄ (µM) | Coastal vs Offshore | 0.817 | 0.250 to 1.384 | 0.002 | Yes |
| PO₄ (µM) | Coastal vs River Mouth | -0.057 | -0.624 to 0.510 | 0.969 | No |
| PO₄ (µM) | Offshore vs River Mouth | -0.874 | -1.441 to -0.307 | 0.001 | Yes |
| SiO₂ (µM) | Coastal vs Offshore | 0.145 | -1.383 to 1.673 | 0.973 | No |
| SiO₂ (µM) | Coastal vs River Mouth | 0.597 | -0.931 to 2.125 | 0.625 | No |
| SiO₂ (µM) | Offshore vs River Mouth | 0.452 | -1.076 to 1.980 | 0.763 | No |
| Chlorophyll-a (µg L⁻¹) | Coastal vs Offshore | -0.040 | -0.744 to 0.663 | 0.990 | No |
| Chlorophyll-a (µg L⁻¹) | Coastal vs River Mouth | -0.352 | -1.056 to 0.352 | 0.464 | No |
| Chlorophyll-a (µg L⁻¹) | Offshore vs River Mouth | -0.312 | -1.016 to 0.392 | 0.547 | No |
| Temperature (°C) | Coastal vs Offshore | -0.436 | -3.338 to 2.467 | 0.933 | No |
| Temperature (°C) | Coastal vs River Mouth | 0.307 | -2.595 to 3.210 | 0.966 | No |
| Temperature (°C) | Offshore vs River Mouth | 0.743 | -2.159 to 3.645 | 0.817 | No |
| Salinity | Coastal vs Offshore | 0.098 | -0.090 to 0.285 | 0.437 | No |
| Salinity | Coastal vs River Mouth | -0.146 | -0.334 to 0.042 | 0.160 | No |
| Salinity | Offshore vs River Mouth | -0.243 | -0.431 to -0.056 | 0.007 | Yes |
