# Supplementary material for: Photic zone dynamics and environmental controls of pigment-derived phytoplankton size classes in the South-Eastern Black Sea
Source: J Plankton Res. 2026 Aug 21;48(5):fbag063. doi: 10.1093/plankt/fbag063 (PMC13496243; doi:10.1093/plankt/fbag063)
Supplement: Supplementary file 1 [file supplementary_materials_fbag063.zip › Supplementary_materials_fbag063_Table 5.doc]

**Supplementary Table S5.** One-way ANOVA results for differences among stations (ANOVA tested differences among River Mouth, Coastal, and Offshore stations. df = between-groups, within-groups. * p < 0.05; ** p < 0.01; *** p < 0.001)

| **Variable** | **df** | **F** | **p** | **Significance** | **River Mouth mean ± SD** | **Coastal mean ± SD** | **Offshore mean ± SD** |
| --- | --- | --- | --- | --- | --- | --- | --- |
| Microphytoplankton (%) | 2, 141 | 1.408 | 0.248 |  | 60.976 ± 17.560 | 58.378 ± 18.180 | 54.249 ± 23.200 |
| Nanophytoplankton (%) | 2, 141 | 1.631 | 0.199 |  | 19.569 ± 14.017 | 16.269 ± 10.801 | 20.788 ± 13.020 |
| Picophytoplankton (%) | 2, 141 | 2.279 | 0.106 |  | 19.455 ± 12.057 | 25.353 ± 16.014 | 24.964 ± 16.900 |
| NO₂+NO₃ (µM) | 2, 141 | 1.862 | 0.159 |  | 0.791 ± 0.880 | 0.578 ± 0.657 | 0.541 ± 0.447 |
| PO₄ (µM) | 2, 141 | 8.347 | <0.001 | *** | 0.220 ± 0.425 | 0.276 ± 0.482 | 1.094 ± 1.927 |
| SiO₂ (µM) | 2, 141 | 0.466 | 0.629 |  | 4.230 ± 2.915 | 3.633 ± 3.954 | 3.777 ± 2.415 |
| Chlorophyll-a (µg L⁻¹) | 2, 141 | 0.841 | 0.434 |  | 1.745 ± 1.436 | 2.098 ± 1.531 | 2.057 ± 1.397 |
| Temperature (°C) | 2, 141 | 0.186 | 0.831 |  | 15.945 ± 5.977 | 15.638 ± 6.145 | 15.202 ± 5.883 |
| Salinity (‰) | 2, 141 | 4.783 | 0.010 | ** | 17.859 ± 0.540 | 18.005 ± 0.320 | 18.102 ± 0.241 |
