# Supplementary material for: Photic zone dynamics and environmental controls of pigment-derived phytoplankton size classes in the South-Eastern Black Sea
Source: J Plankton Res. 2026 Aug 21;48(5):fbag063. doi: 10.1093/plankt/fbag063 (PMC13496243; doi:10.1093/plankt/fbag063)
Supplement: Supplementary file 1 [file supplementary_materials_fbag063.zip › Supplementary_materials_fbag063_Table 4.doc]

**Supplementary Table S4.** Pearson correlations between phytoplankton size classes and environmental variables (Values are Pearson r coefficients followed by significance symbols; p values are provided in adjacent columns. * p < 0.05; ** p < 0.01; *** p < 0.001. Overall analysis includes all stations and depths (n = 144).

| **Environmental variables** | **Microphytoplankton (%)** | | **Nanophytoplankton (%)** | | **Picophytoplankton (%)** | |
| --- | --- | --- | --- | --- | --- | --- |
|  | **r** | **p** | **r** | **p** | **r** | **p** |
| NO₂+NO₃ (µM) | 0.093 | 0.268 | -0.094 | 0.263 | -0.043 | 0.613 |
| PO₄ (µM) | 0.082 | 0.329 | -0.098 | 0.241 | -0.025 | 0.770 |
| SiO₂ (µM) | -0.057 | 0.498 | 0.186* | 0.026 | -0.081 | 0.335 |
| Chl-*a* (µg L⁻¹) | 0.489*** | <0.001 | -0.309*** | <0.001 | -0.378*** | <0.001 |
| Temperature (°C) | -0.016 | 0.844 | 0.022 | 0.790 | 0.003 | 0.973 |
| Salinity (‰) | -0.060 | 0.474 | 0.004 | 0.964 | 0.075 | 0.371 |
