# Supplementary material for: Photic zone dynamics and environmental controls of pigment-derived phytoplankton size classes in the South-Eastern Black Sea
Source: J Plankton Res. 2026 Aug 21;48(5):fbag063. doi: 10.1093/plankt/fbag063 (PMC13496243; doi:10.1093/plankt/fbag063)
Supplement: Supplementary file 1 [file supplementary_materials_fbag063.zip › Supplementary_materials_fbag063_Table 3.doc]

Supplementary Table 3. HPLC pigment concentrations measured at the Offshore station (detection limit =0.005 µg/l)

| **Season** | **Depth** | **Perid** | **19-But** | **Fuco** | **19-Hex** | **Allo** | **Zea** | **Chl-*b*** | **Chl-*a*** |
| --- | --- | --- | --- | --- | --- | --- | --- | --- | --- |
| May | Surface | <0.005 | <0.005 | 0.05 | <0.005 | <0.005 | 0.18 | 0.26 | 1.24 |
|  | 10 | <0.005 | <0.005 | 0.08 | <0.005 | <0.005 | 0.26 | 0.22 | 1.10 |
|  | 25 | <0.005 | <0.005 | 0.18 | <0.005 | <0.005 | 0.40 | 0.34 | 2.11 |
|  | 50 | <0.005 | <0.005 | 0.25 | <0.005 | <0.005 | 0.35 | 0.18 | 1.82 |
| June | Surface | <0.005 | <0.005 | 0.04 | 0.18 | <0.005 | <0.005 | 0.14 | 0.53 |
|  | 10 | 0.20 | <0.005 | 0.12 | 0.32 | <0.005 | <0.005 | 0.15 | 0.96 |
|  | 25 | 0.47 | <0.005 | 0.24 | 0.30 | <0.005 | <0.005 | 0.20 | 1.44 |
|  | 50 | <0.005 | <0.005 | 0.22 | 0.32 | <0.005 | <0.005 | 0.22 | 0.52 |
| July | Surface | 0.11 | <0.005 | 0.03 | 0.15 | <0.005 | 0.22 | <0.005 | 0.76 |
|  | 10 | 0.24 | <0.005 | 0.06 | 0.19 | <0.005 | 0.36 | <0.005 | 0.91 |
|  | 25 | 0.30 | <0.005 | 0.30 | 0.37 | <0.005 | 1.21 | <0.005 | 2.24 |
|  | 50 | <0.005 | <0.005 | 0.05 | 0.05 | <0.005 | 0.16 | 0.18 | 0.64 |
| August | Surface | 0.17 | <0.005 | 0.20 | 0.18 | <0.005 | 0.47 | <0.005 | 1.09 |
|  | 10 | 0.83 | <0.005 | 0.08 | 0.30 | <0.005 | 0.72 | <0.005 | 0.67 |
|  | 25 | 1.76 | <0.005 | 1.06 | 0.42 | 0.05 | 0.39 | 0.28 | 3.23 |
|  | 50 | 1.22 | <0.005 | 1.20 | 0.27 | 0.11 | 0.50 | 0.41 | 3.93 |
| September | Surface | 1.01 | <0.005 | 0.39 | 0.48 | 0.05 | <0.005 | 0.18 | 2.35 |
|  | 10 | 0.71 | <0.005 | 0.43 | 0.08 | 0.06 | <0.005 | 0.11 | 2.19 |
|  | 25 | 1.12 | <0.005 | 1.05 | 0.27 | 0.09 | <0.005 | 0.24 | 3.19 |
|  | 50 | 0.32 | <0.005 | 0.55 | 0.15 | 0.06 | <0.005 | 0.08 | 2.23 |
| October | Surface | <0.005 | <0.005 | 0.68 | 0.43 | <0.005 | 0.64 | 0.38 | 2.00 |
|  | 10 | <0.005 | <0.005 | 0.28 | 0.10 | <0.005 | 0.14 | <0.005 | 0.57 |
|  | 25 | 0.36 | <0.005 | 0.62 | 0.09 | <0.005 | 0.33 | 0.60 | 3.47 |
|  | 50 | <0.005 | <0.005 | 0.03 | 0.04 | <0.005 | 0.06 | <0.005 | 0.21 |
| November | Surface | 0.39 | <0.005 | 0.13 | 0.14 | <0.005 | 0.28 | <0.005 | 0.40 |
|  | 10 | 0.45 | <0.005 | 0.14 | 0.23 | <0.005 | 0.31 | 0.18 | 1.11 |
|  | 25 | 0.47 | <0.005 | 0.29 | 0.27 | <0.005 | 0.93 | <0.005 | 2.13 |
|  | 50 | 0.20 | <0.005 | 0.18 | 0.22 | <0.005 | 0.41 | <0.005 | 0.28 |
| December | Surface | 0.80 | <0.005 | 0.78 | <0.005 | 0.12 | 0.20 | 0.16 | 4.13 |
|  | 10 | 0.55 | <0.005 | 0.86 | 0.11 | 0.12 | 0.27 | <0.005 | 4.31 |
|  | 25 | 1.36 | 0.56 | 2.45 | 1.76 | 1.53 | 0.58 | 3.60 | 4.01 |
|  | 50 | 1.66 | 0.55 | 0.10 | 0.38 | 0.16 | <0.005 | 0.38 | 4.33 |
| January | Surface | 0.56 | <0.005 | 0.12 | 0.09 | 0.09 | 0.15 | 0.15 | 0.72 |
|  | 10 | 0.24 | 0.09 | 0.22 | 0.15 | 0.13 | 0.21 | 0.45 | 1.69 |
|  | 25 | <0.005 | <0.005 | 0.21 | 0.05 | 0.06 | 0.14 | 0.16 | 1.48 |
|  | 50 | 0.22 | <0.005 | 0.18 | 0.04 | 0.03 | 0.14 | <0.005 | 1.03 |
| February | Surface | <0.005 | <0.005 | 0.23 | 0.14 | 0.11 | 0.15 | 0.39 | 1.99 |
|  | 10 | 0.17 | <0.005 | 0.23 | 0.10 | 0.09 | 0.11 | 0.34 | 2.31 |
|  | 25 | 0.24 | <0.005 | 0.18 | 0.05 | 0.11 | 0.07 | 0.38 | 1.86 |
|  | 50 | <0.005 | <0.005 | 0.16 | 0.06 | 0.06 | 0.07 | 0.27 | 1.02 |
| March | Surface | 0.70 | <0.005 | 0.82 | 0.29 | 0.07 | 0.19 | 0.18 | 2.97 |
|  | 10 | 0.48 | <0.005 | 2.61 | 0.23 | 0.13 | 0.51 | 0.52 | 3.41 |
|  | 25 | 0.28 | <0.005 | 1.31 | 0.35 | <0.005 | 0.09 | 0.48 | 2.86 |
|  | 50 | 0.39 | <0.005 | 2.14 | 0.40 | 0.09 | 0.27 | 0.52 | 2.45 |
| April | Surface | <0.005 | <0.005 | 0.14 | 0.30 | 0.02 | <0.005 | 0.11 | 1.06 |
|  | 10 | <0.005 | <0.005 | 0.15 | 0.16 | <0.005 | <0.005 | 0.09 | 0.76 |
|  | 25 | <0.005 | <0.005 | 0.58 | 0.21 | <0.005 | <0.005 | 0.08 | 1.45 |
|  | 50 | <0.005 | <0.005 | 0.13 | 0.22 | <0.005 | <0.005 | 0.13 | 0.54 |
