# Supplementary material for: Photic zone dynamics and environmental controls of pigment-derived phytoplankton size classes in the South-Eastern Black Sea
Source: J Plankton Res. 2026 Aug 21;48(5):fbag063. doi: 10.1093/plankt/fbag063 (PMC13496243; doi:10.1093/plankt/fbag063)
Supplement: Supplementary file 1 [file supplementary_materials_fbag063.zip › Supplementary_materials_fbag063_Table 2.doc]

Supplementary Table 2. HPLC pigment concentrations measured at the Coastal station (detection limit =0.005 µg/l)

| **Season** | **Depth** | **Perid** | **19-But** | **Fuco** | **19-Hex** | **Allo** | **Zea** | **Chl-*b*** | **Chl-*a*** |
| --- | --- | --- | --- | --- | --- | --- | --- | --- | --- |
| May | Surface | 0.72 | <0.005 | 0.68 | 0.61 | <0.005 | 0.45 | 0.43 | 3.55 |
|  | 10 | <0.005 | <0.005 | 0.09 | 0.07 | <0.005 | <0.005 | 0.33 | 0.66 |
|  | 25 | 0.47 | <0.005 | 0.46 | 0.41 | <0.005 | 0.38 | 0.44 | 3.03 |
|  | 50 | <0.005 | <0.005 | 0.36 | 0.42 | <0.005 | <0.005 | 0.54 | 0.07 |
| June | Surface | 0.46 | <0.005 | 0.07 | 0.30 | <0.005 | 0.19 | <0.005 | 0.56 |
|  | 10 | 0.65 | <0.005 | 0.20 | 0.61 | 0.03 | 0.21 | 0.29 | 1.89 |
|  | 25 | 0.89 | 0.04 | 0.63 | 0.44 | 0.05 | 0.29 | <0.005 | 3.04 |
|  | 50 | 0.99 | <0.005 | 0.07 | 0.45 | 0.04 | 0.08 | <0.005 | 0.39 |
| July | Surface | 0.14 | <0.005 | 0.04 | 0.13 | <0.005 | 0.13 | <0.005 | 0.52 |
|  | 10 | 0.20 | <0.005 | 0.09 | 0.07 | <0.005 | 0.26 | <0.005 | 0.63 |
|  | 25 | 0.92 | 0.04 | 0.16 | 0.35 | 0.08 | 0.40 | 0.21 | 2.73 |
|  | 50 | 0.62 | <0.005 | 0.23 | <0.005 | 0.02 | 0.38 | 0.32 | 1.90 |
| August | Surface | <0.005 | <0.005 | 0.33 | 0.08 | <0.005 | 0.55 | <0.005 | 1.06 |
|  | 10 | <0.005 | <0.005 | 0.17 | 0.13 | <0.005 | 0.36 | <0.005 | 0.97 |
|  | 25 | 2.64 | 0.09 | 1.68 | 0.62 | 0.05 | 0.44 | <0.005 | 4.99 |
|  | 50 | 1.61 | <0.005 | 1.30 | 0.23 | <0.005 | 0.39 | <0.005 | 4.30 |
| September | Surface | <0.005 | <0.005 | 0.19 | 0.04 | <0.005 | 0.27 | <0.005 | 0.36 |
|  | 10 | 0.30 | <0.005 | 0.13 | 0.06 | <0.005 | 0.42 | <0.005 | 0.84 |
|  | 25 | 0.21 | <0.005 | 0.11 | 0.12 | 0.04 | 0.22 | 0.40 | 2.56 |
|  | 50 | <0.005 | <0.005 | 0.20 | 0.16 | <0.005 | 0.14 | 0.10 | 1.01 |
| October | Surface | 0.83 | <0.005 | 3.34 | 0.38 | 0.09 | 0.19 | 1.10 | 4.13 |
|  | 10 | 0.30 | <0.005 | 2.33 | 0.17 | 0.02 | 0.06 | 0.34 | 1.51 |
|  | 25 | 0.44 | <0.005 | 1.43 | 0.04 | <0.005 | 0.09 | 0.12 | 0.98 |
|  | 50 | 0.47 | <0.005 | 0.10 | 0.16 | <0.005 | 0.07 | <0.005 | 0.68 |
| November | Surface | 1.71 | <0.005 | 1.30 | 0.42 | <0.005 | 0.31 | 0.74 | 5.67 |
|  | 10 | 1.27 | <0.005 | 1.60 | 1.12 | <0.005 | <0.005 | 1.30 | 4.07 |
|  | 25 | 0.60 | <0.005 | 0.71 | 0.28 | 0.08 | <0.005 | 0.54 | 3.94 |
|  | 50 | 0.39 | <0.005 | 0.61 | 0.20 | 0.08 | <0.005 | 0.20 | 2.59 |
| December | Surface | 1.91 | 0.16 | 0.27 | 0.33 | 0.24 | 0.79 | 0.52 | 3.07 |
|  | 10 | 0.72 | <0.005 | 0.37 | 0.16 | 0.21 | 0.62 | 0.79 | 2.61 |
|  | 25 | 0.84 | 0.03 | 0.62 | 1.06 | 0.18 | 0.48 | 0.41 | 2.66 |
|  | 50 | 0.67 | <0.005 | 0.24 | 0.10 | 0.04 | 0.30 | 0.23 | 2.54 |
| January | Surface | <0.005 | <0.005 | 0.07 | 0.07 | 0.03 | 0.13 | <0.005 | 0.55 |
|  | 10 | 0.27 | <0.005 | 0.13 | 0.04 | 0.06 | 0.18 | 0.16 | 1.27 |
|  | 25 | <0.005 | <0.005 | 0.27 | 0.09 | 0.10 | 0.21 | 0.26 | 1.81 |
|  | 50 | <0.005 | <0.005 | 0.14 | 0.04 | <0.005 | 0.11 | <0.005 | 0.80 |
| February | Surface | 0.89 | <0.005 | 0.06 | 0.14 | 0.04 | 0.06 | 0.40 | 1.65 |
|  | 10 | <0.005 | <0.005 | 0.13 | 0.03 | 0.04 | 0.12 | 0.23 | 1.14 |
|  | 25 | <0.005 | <0.005 | 0.06 | <0.005 | 0.02 | 0.09 | 0.14 | 0.74 |
|  | 50 | 0.32 | <0.005 | 0.14 | 0.07 | 0.05 | 0.21 | 0.31 | 1.42 |
| March | Surface | 0.70 | <0.005 | 1.16 | 0.40 | 0.07 | 0.12 | 0.30 | 0.97 |
|  | 10 | 0.77 | <0.005 | 1.18 | 0.10 | 0.04 | 0.15 | 0.41 | 3.07 |
|  | 25 | 0.32 | <0.005 | 2.91 | 0.15 | 0.04 | 0.19 | 0.25 | 5.40 |
|  | 50 | 0.41 | <0.005 | 1.79 | 0.17 | <0.005 | 0.11 | 0.35 | 4.19 |
| April | Surface | 0.56 | <0.005 | 0.60 | 0.98 | 0.38 | <0.005 | 0.20 | 4.90 |
|  | 10 | <0.005 | <0.005 | 0.48 | 0.47 | 0.04 | <0.005 | 0.18 | 1.91 |
|  | 25 | <0.005 | <0.005 | 0.35 | 0.03 | 0.06 | 0.05 | 0.16 | 1.27 |
|  | 50 | <0.005 | <0.005 | 0.19 | 0.12 | 0.03 | <0.005 | 0.23 | 0.08 |
