# Supplementary material for: Photic zone dynamics and environmental controls of pigment-derived phytoplankton size classes in the South-Eastern Black Sea
Source: J Plankton Res. 2026 Aug 21;48(5):fbag063. doi: 10.1093/plankt/fbag063 (PMC13496243; doi:10.1093/plankt/fbag063)
Supplement: Supplementary file 1 [file supplementary_materials_fbag063.zip › Supplementary_materials_fbag063_Table 1.doc]

Supplementary Table 1. HPLC pigment concentrations measured at the River Mouth station (detection limit =0.005 µg/l)

| **Season** | **Depth** | **Perid** | **19-But** | **Fuco** | **19-Hex** | **Allo** | **Zea** | **Chl-*b*** | **Chl-*a*** |
| --- | --- | --- | --- | --- | --- | --- | --- | --- | --- |
| May | Surface | 0.88 | <0.005 | 0.55 | 0.31 | <0.005 | 0.24 | 0.21 | 2.92 |
|  | 10 | <0.005 | <0.005 | 0.23 | 0.26 | <0.005 | 0.11 | 0.09 | 1.10 |
|  | 25 | <0.005 | <0.005 | 0.33 | 0.22 | <0.005 | 0.25 | 0.13 | 1.84 |
|  | 50 | <0.005 | <0.005 | 0.20 | 0.27 | <0.005 | 0.28 | 0.15 | 0.21 |
| June | Surface | 2.29 | <0.005 | 0.62 | 0.98 | 0.15 | 0.13 | 0.19 | 6.15 |
|  | 10 | 1.45 | <0.005 | 1.02 | 0.92 | 0.09 | 0.04 | 0.21 | 6.32 |
|  | 25 | 0.63 | <0.005 | 0.27 | 0.14 | <0.005 | 0.04 | <0.005 | 0.98 |
|  | 50 | <0.005 | <0.005 | 0.06 | 0.08 | <0.005 | 0.06 | 0.12 | 0.69 |
| July | Surface | 0.44 | <0.005 | 0.32 | 0.54 | 0.12 | 0.18 | 0.15 | 2.22 |
|  | 10 | 0.35 | <0.005 | 0.40 | 0.39 | 0.08 | 0.14 | <0.005 | 2.18 |
|  | 25 | 0.31 | <0.005 | 0.93 | 0.28 | 0.10 | 0.17 | <0.005 | 2.73 |
|  | 50 | 0.26 | <0.005 | 0.46 | 0.30 | 0.06 | 0.13 | <0.005 | 0.21 |
| August | Surface | <0.005 | <0.005 | 0.50 | 0.07 | <0.005 | 0.74 | <0.005 | 1.91 |
|  | 10 | <0.005 | <0.005 | 0.38 | 0.14 | <0.005 | 0.55 | <0.005 | 1.62 |
|  | 25 | 0.91 | <0.005 | 0.52 | 0.32 | <0.005 | 0.15 | 0.08 | 2.30 |
|  | 50 | <0.005 | <0.005 | 0.41 | 0.22 | <0.005 | 0.18 | 0.09 | 1.70 |
| September | Surface | <0.005 | <0.005 | 0.31 | 0.15 | <0.005 | 0.37 | 0.15 | 0.81 |
|  | 10 | 0.38 | <0.005 | 0.30 | 0.09 | <0.005 | 0.31 | <0.005 | 0.73 |
|  | 25 | <0.005 | <0.005 | 0.23 | 0.07 | <0.005 | 0.25 | <0.005 | 0.37 |
|  | 50 | <0.005 | <0.005 | 0.04 | 0.04 | <0.005 | 0.05 | <0.005 | 0.28 |
| October | Surface | 0.51 | <0.005 | 2.28 | 0.13 | 0.04 | 0.07 | 0.37 | 0.83 |
|  | 10 | 0.75 | <0.005 | 2.94 | 0.14 | 0.15 | 0.09 | 2.13 | 3.18 |
|  | 25 | 0.61 | <0.005 | 1.61 | 0.19 | <0.005 | 0.09 | <0.005 | 0.37 |
|  | 50 | 0.48 | <0.005 | 0.10 | 0.22 | <0.005 | 0.10 | <0.005 | 0.40 |
| November | Surface | 1.14 | <0.005 | 1.71 | 0.40 | <0.005 | <0.005 | 0.46 | 3.19 |
|  | 10 | 1.38 | <0.005 | 2.15 | 0.62 | <0.005 | <0.005 | 0.61 | 4.24 |
|  | 25 | 0.73 | <0.005 | 0.33 | 0.14 | <0.005 | <0.005 | 0.25 | 2.10 |
|  | 50 | <0.005 | <0.005 | 0.16 | 0.12 | <0.005 | <0.005 | 0.09 | 0.38 |
| December | Surface | 0.51 | <0.005 | 0.52 | 0.06 | 0.07 | 0.29 | 0.19 | 3.28 |
|  | 10 | 0.48 | <0.005 | 0.12 | 0.09 | 0.06 | 0.23 | 0.13 | 3.74 |
|  | 25 | <0.005 | <0.005 | 0.06 | 0.16 | 0.04 | 0.03 | <0.005 | 1.30 |
|  | 50 | <0.005 | <0.005 | 0.24 | 0.22 | <0.005 | 0.15 | <0.005 | 1.19 |
| January | Surface | 0.38 | <0.005 | 0.14 | 0.11 | 0.06 | 0.25 | 0.15 | 1.36 |
|  | 10 | 0.66 | <0.005 | 0.28 | 0.09 | 0.08 | 0.24 | 0.30 | 1.97 |
|  | 25 | 0.31 | <0.005 | 0.18 | 0.07 | 0.04 | 0.09 | 0.19 | 0.94 |
|  | 50 | 0.37 | <0.005 | 0.10 | 0.09 | <0.005 | 0.13 | 0.22 | 0.37 |
| February | Surface | 0.25 | <0.005 | 0.09 | 0.06 | 0.10 | 0.06 | 0.17 | 1.02 |
|  | 10 | 0.19 | <0.005 | 0.26 | 0.16 | 0.09 | 0.10 | 0.31 | 1.44 |
|  | 25 | 0.27 | <0.005 | 0.15 | 0.06 | 0.05 | 0.13 | 0.18 | 1.15 |
|  | 50 | 0.41 | <0.005 | 0.05 | 0.11 | <0.005 | 0.06 | 0.28 | 0.55 |
| March | Surface | 0.51 | <0.005 | 0.74 | 0.06 | 0.09 | 0.15 | 0.26 | 2.02 |
|  | 10 | 0.32 | <0.005 | 1.82 | 0.07 | <0.005 | <0.005 | 0.20 | 3.11 |
|  | 25 | 0.68 | <0.005 | 2.75 | 0.05 | 0.02 | <0.005 | 0.43 | 3.77 |
|  | 50 | 0.56 | <0.005 | 0.25 | 0.09 | <0.005 | <0.005 | 0.44 | 0.45 |
| April | Surface | <0.005 | <0.005 | 0.32 | 0.52 | 0.03 | 0.10 | <0.005 | 2.00 |
|  | 10 | <0.005 | <0.005 | 0.39 | 0.33 | <0.005 | 0.14 | <0.005 | 1.46 |
|  | 25 | <0.005 | <0.005 | 0.41 | 0.14 | <0.005 | 0.08 | <0.005 | 0.14 |
|  | 50 | <0.005 | <0.005 | 0.24 | 0.17 | <0.005 | 0.10 | 0.08 | 0.56 |
